# Electrolyte Li^+^ Chemical Potential Correlates with Graphite Negative Electrode Reactions in Lithium‐Ion Batteries

**DOI:** 10.1002/adma.202514060

**Published:** 2025-10-25

**Authors:** Yasuyuki Kondo, Haruna Nakajima, Yu Katayama, Nao Kobayashi, Shinya Otani, Akinori Tani, Shigeaki Yamazaki, Yuki Yamada

**Affiliations:** ^1^ SANKEN The University of Osaka Ibaraki Osaka 567‐0047 Japan; ^2^ Chemicals Division Daikin Industries, Ltd. Settsu Osaka 566‐8585 Japan

**Keywords:** electrolyte lithium‐ion chemical potential, graphite negative electrodes, lithium‐ion batteries, solvation environment, solvent cointercalation

## Abstract

Novel electrolytes for advanced lithium‐ion batteries (LIBs) with higher energy density and safety are being extensively explored. A major challenge in developing new electrolytes is achieving reversible Li^+^ intercalation into graphite negative electrodes. In commercial LIBs, this reaction is reversible in ethylene carbonate (EC) electrolytes, whereas unfavorable Li^+^–solvent cointercalation occurs in many other electrolytes. Recently, EC‐free Li^+^ intercalation has been achieved in some types of advanced electrolytes, including (localized) highly concentrated electrolytes and weakly coordinating electrolytes. However, an essential factor that dominates whether Li^+^ intercalation or Li^+^–solvent cointercalation occurs has yet to be identified. Herein, the electrolyte Li^+^ chemical potential is reported as a quantitative descriptor of the Li^+^ intercalation behavior. Solvent cointercalation is generally inhibited above a certain threshold of the electrolyte Li^+^ chemical potential. This work provides a novel guideline for designing advanced LIB electrolytes.

## Graphical abstract

This work demonstrates that the electrolyte Li^+^ chemical potential (*µ*_Li_^+^) is a quantitative descriptor of electrochemical Li^+^ intercalation into graphite negative electrodes. Solvent cointercalation can be generally suppressed, and Li^+^ intercalation is enabled by employing electrolytes with t high *µ*_Li_^+^ exceeding the boundary region, which provides a design principle for advanced battery electrolytes.

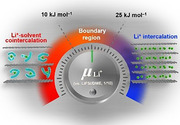

## 1 Introduction

Lithium‐ion batteries (LIBs) have been adopted as the main power supplies in electronic devices over the past few decades. Recently, there has been a growing demand for both higher performance and higher safety toward large‐scale applications.^[^ ^1^ ^]^ Currently, the performance and safety are limited by state‐of‐the‐art ethylene carbonate (EC) electrolytes.^[^ ^2^, ^3^, ^4^, ^5^ ^]^ EC is known as an essential solvent for forming a stable solid electrolyte interphase (SEI) on graphite negative electrodes, which effectively suppresses side reactions (e.g., electrolyte reduction) and enables reversible Li^+^ intercalation during charge and discharge.^[^ ^6^ ^]^ To overcome the limits of LIBs, various alternative solvents with higher oxidation stability, stronger Lewis basicity, lower melting point, and/or nonflammability have been examined.^[^ ^7^, ^8^, ^9^ ^]^ In most cases, a solvent molecule is unfortunately cointercalated into graphite in the form of solvated Li^+^, which is a destructive side reaction that leads to lower capacities and/or poor cycling stability.^[^ ^10^ ^]^

**Figure 1:**
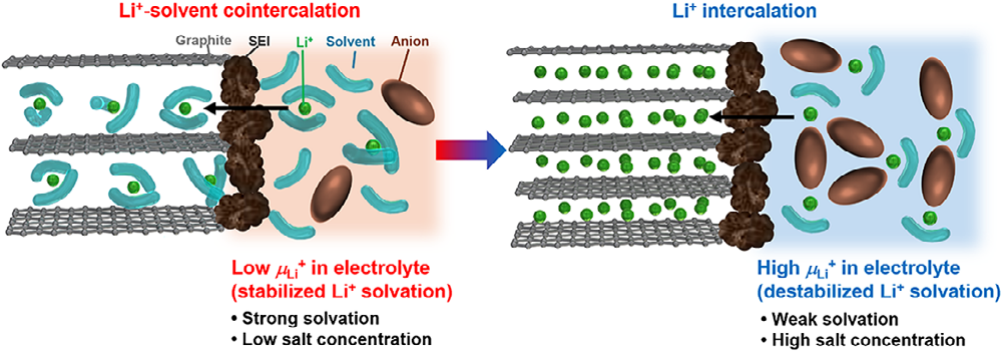
Schematics of graphite negative electrode reactions in various electrolytes depending on the electrolyte Li^+^ chemical potential *µ*_Li_^+^. Left: Li^+^‐solvent cointercalation proceeds in electrolytes with a low *µ*_Li_^+^. Right: Li^+^ successfully intercalates into graphite in electrolytes with high *µ*_Li_^+^. Graphite electrode reactions more strongly correlate with *µ_L_*_i_^+^ than the nature of SEI.

To prevent unfavorable solvent cointercalation, several strategies have been proposed, for example, employing highly concentrated electrolytes, localized highly concentrated electrolytes,^[^ ^11^, ^12^, ^13^, ^14^, ^15^, ^16^, ^17^ ^]^ and weakly coordinating electrolytes (**Figure** **1**).^[^ ^18^, ^19^, ^20^, ^21^, ^22^, ^23^, ^24^ ^]^ Specifically, lithium bis(fluorosulfonyl)imide (LiFSI) is widely used in these electrolytes, in which the formation of unique FSI anion‐derived SEIs on graphite, induced by preferential anion coordination to Li^+^, is believed to suppress solvent cointercalation.^[^ ^13^, ^25^, ^26^ ^]^ However, no quantitative relationship has been identified between the SEI chemistry and suppressed solvent cointercalation. Some publications have noted other dominant factors in addition to the SEI, such as the solvation structure^[^ ^27^, ^28^ ^]^ and free solvent activity.^[^ ^29^ ^]^ Nevertheless, no quantitative factor that can accurately describe the relationship between the intrinsic properties of electrolytes and Li^+^ intercalation reactions has been discovered. As a result, the exploration of new electrolytes still depends on trial and error without a firm scientific foundation.

Herein, we report the Li^+^ chemical potential (*µ*_Li_^+^) in electrolytes as a quantitative factor that strongly correlates with the Li^+^ intercalation behavior of graphite negative electrodes. By definition, *µ*_Li_^+^ is the partial molar Gibbs free energy of Li^+^, which quantitatively describes the thermodynamic stability of Li^+^ in the electrolyte. Specifically, higher (lower) *µ*_Li_^+^ means unstable (stable) Li^+^ in the solvation environment. In our previous work, we reported that *µ*_Li_^+^ is commonly increased (thus, Li^+^ is destabilized) in (localized) highly concentrated electrolytes and weakly coordinating electrolytes.^[^ ^30^ ^]^ Based on this intuition, here, we scrutinized the correlation between *µ*_Li_^+^ and the Li^+^ intercalation behavior in various electrolytes (Figure 1), and discovered threshold *µ*_Li_^+^ above which Li^+^ intercalation is achieved without solvent cointercalation.

## 2 Results and Discussion

### 2.1 Variation in *µ*_Li_^+^ Depending on Electrolyte Structure

Various organic electrolytes were prepared by mixing LiFSI and organic solvents with different solvation abilities (Figure S1, Supporting Information). The *µ*_Li_^+^ values were evaluated by measuring the lithium electrode potential (*E*_Li_) with reference to the redox potential of ferrocene/ferrocenium (Fc/Fc^+^) as an internal standard^[^ ^31^ ^]^ (Figure S2, Supporting Information) according to the following equation (Note S1, Supporting Information)^[^ ^30^ ^]^:

(1) $$\mu_{Li^{+}}=FE_{\mathrm{Li}}+\mathrm{constant}$$

where *F* denotes the Faraday constant. The evaluated *E*_Li_ and *µ*_Li_^+^ values in various electrolytes are shown in Tables S1–S3 (Supporting Information). Herein, *µ*_Li_^+^ is shown as a relative value to that in LiFSI/1,2‐dimethoxyethane (DME) (1/10 by mol).

**Figure 2:**
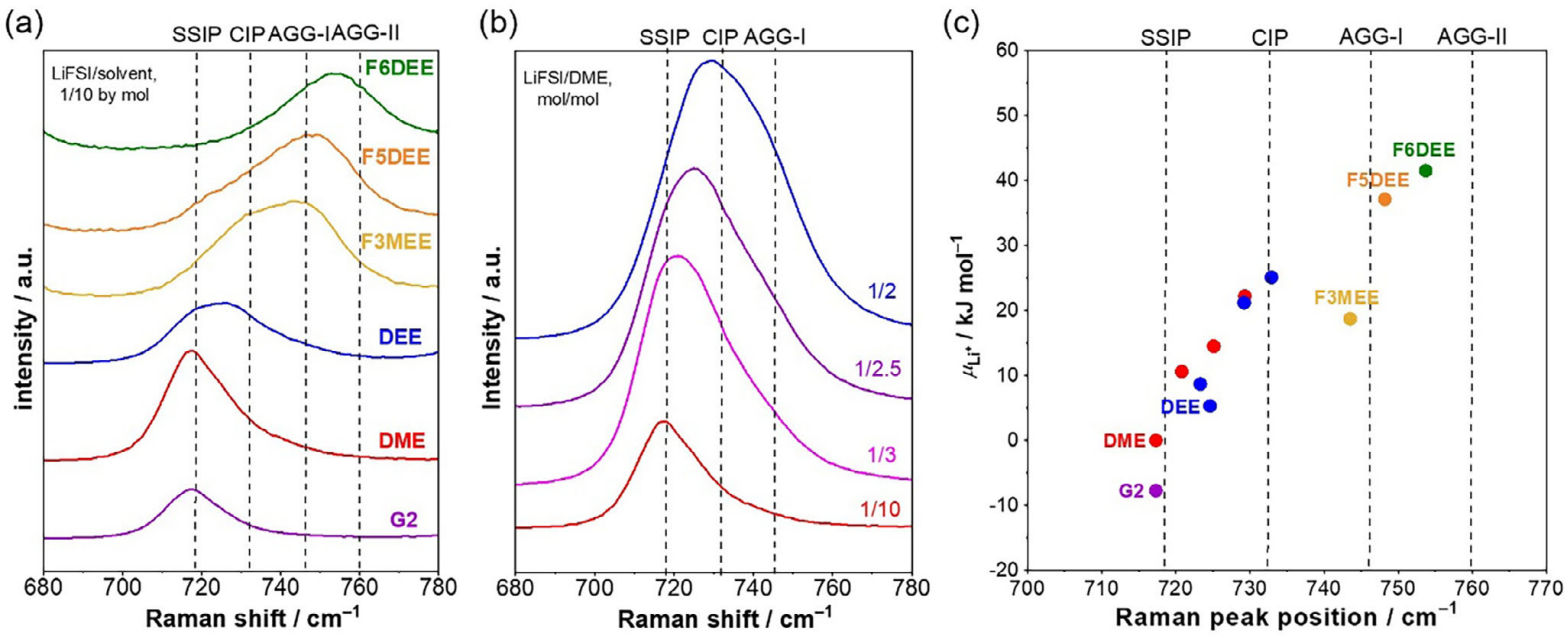
a) Raman spectra of LiFSI/solvent (1/10 by mol) solutions with different solvents (G2, DME, DEE, F3MEE, F5DEE, and F6DEE). b) Raman spectra of LiFSI/DME solutions at different salt/solvent molar ratios (1/10, 1/3, 1/2.5, and 1/2). The Raman band between 680 and 780 cm^−1^ is derived from the vibration of FSI^−^. The peak position represents the ion pairing states of Li^+^ and FSI^−^, namely SSIP, CIP, AGG‐I, and AGG‐II.^[^ ^30^, ^32^ ^]^ The peak at a higher wavenumber indicates more extensive ion pairs. c) Electrolyte Li^+^ chemical potential *µ*_Li_^+^ (vs LiFSI/DME, 1/10) of various electrolytes plotted against the Raman peak positions of the FSI^−^ vibration. For DME and DEE, data with various salt concentrations are plotted.

Overall, *µ*_Li_^+^ increases (thus, Li^+^ is destabilized) under the conditions of i) a weaker solvation ability of solvents and ii) a higher salt concentration (including a localized high‐concentration state) as demonstrated in **Figure** **2**. Figure 2a shows the Raman spectra of FSI^−^ vibration in LiFSI electrolytes with various solvents (1/10 by mol). The peak position represents the ion pairing states of Li^+^ and FSI^−^, namely solvent separate ion pair (SSIP), contact ion pair (CIP), and aggregate (AGG‐I and AGG‐II).^[^ ^30^, ^32^ ^]^ The ion pairing becomes more extensive in the order of diglyme (G2) < DME < 1,2‐diethoxyethane (DEE) < 1,1,1‐trifluoro‐2‐(2‐methoxyehoxy) ethane (F3MEE) < 2‐[2‐(2,2‐difluoroethoxy)ethoxy]‐1,1,1‐trifluoroethane (F5DEE) < 1,2‐bis(2,2,2‐trifluoroehoxy) ethane (F6DEE), suggesting that the solvation abilities are in the order of G2 > DME > DEE > F3MEE > F5DEE > F6DEE, which is consistent with theoretical calculations (Table S4, Figure S3, Supporting Information) and previous literature.^[^ ^33^ ^]^ Notably, this order is the same as that for *µ*_Li_^+^, namely G2 (−7.72 kJ mol^−1^) < DME (0 kJ mol^−1^) < DEE (5.31 kJ mol^−1^) < F3MEE (18.7 kJ mol^−1^) < F5DEE (37.1 kJ mol^−1^) < F6DEE (41.5 kJ mol^−1^) (Table S1, Supporting Information). Hence, weaker solvation (i.e., more extensive ion pairing) increases the *µ*_Li_^+^. The same tendency of ion pairing was observed when salt concentrations were increased (Figure 2b; Figure S4, Supporting Information), which also increased the *µ*_Li_^+^, namely LiFSI/DME 1/10 (0 kJ mol^−1^) < 1/3 (10.6 kJ mol^−1^) < 1/2.5 (14.5 kJ mol^−1^) < 1/2 (22.2 kJ mol^−1^). To corroborate the importance of ion pairing, the *µ*_Li_^+^ values in various LiFSI electrolytes with different solvents and salt concentrations are plotted versus the Raman peak positions of FSI^−^ vibration (Figure 2c). As a result, there is a linear correlation between the *µ*_Li_^+^ values and the Raman peak positions, suggesting that the increase in *µ*_Li_^+^ is commonly related to extensive ion pairing of Li^+^ and FSI^−^. In summary, *µ*_Li_^+^ in electrolytes is predominantly determined by the interaction between Li^+^ and anion. Hence, the coordination number (i.e., the extent of ion pairing) and the Lewis basicity of the anion are the important factors to control the *µ*_Li_^+^.

### 2.2 Correlation between *µ*_Li_^+^ and Graphite Negative Electrode Reactions

**Figure 3:**
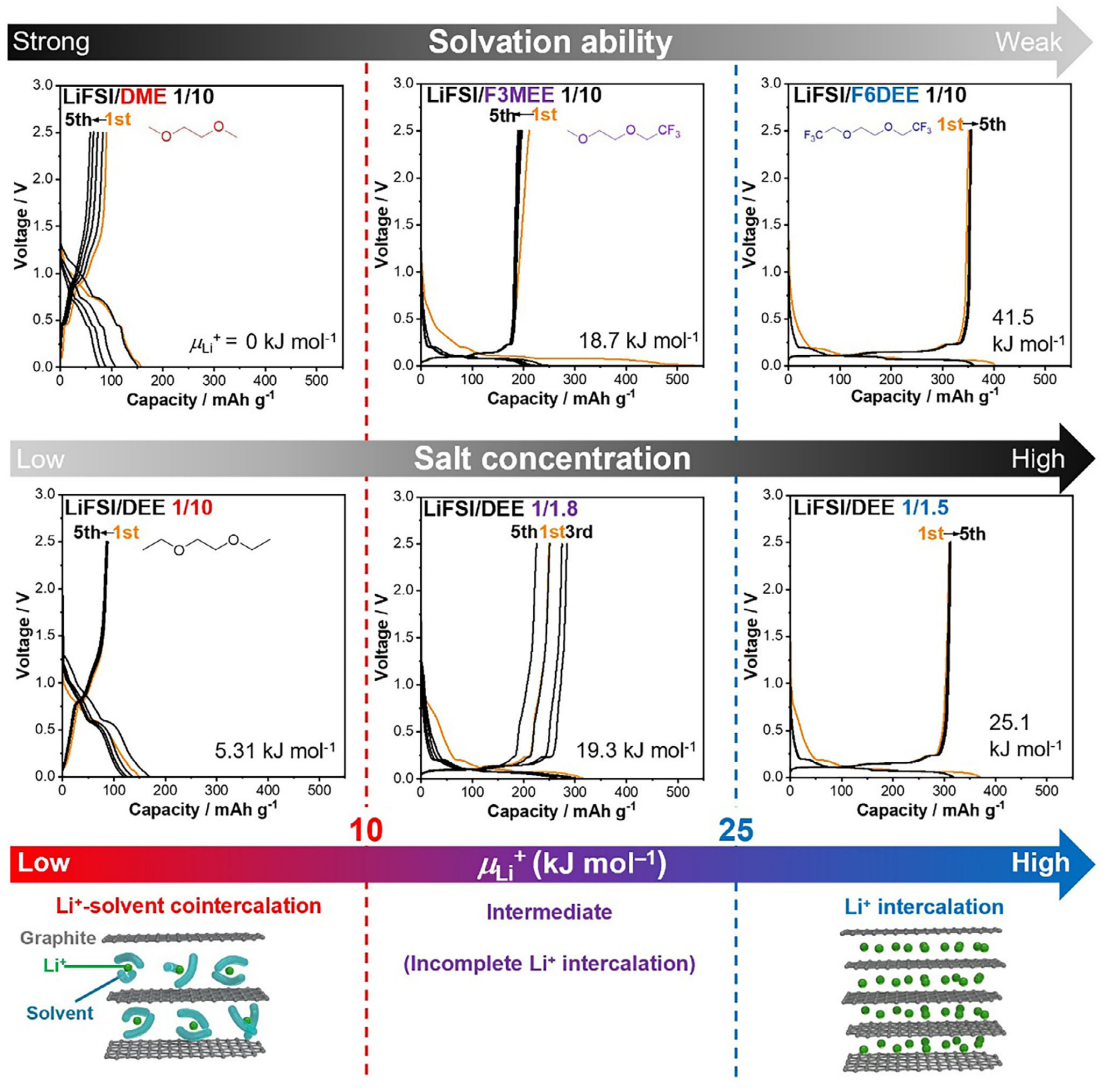
Charge–discharge curves of natural graphite|Li half cells and Li^+^ intercalation mechanisms in LiFSI/DME (1/10 by mol), LiFSI/F3MEE (1/10 by mol), LiFSI/F6DEE (1/10 by mol) and LiFSI/DEE (1/10, 1/1.8 and 1/1.5 by mol) at a C/10 rate (37.2 mA g^−1^) depending on the electrolyte Li^+^ chemical potential *µ*_Li_^+^ (vs LiFSI/DME, 1/10).

Here, we focus on the reactions of graphite in LiFSI electrolytes (LiFSI/solvent = 1/10 by mol) with different solvation abilities and* µ*_Li_^+^ values, namely, DME (0 kJ mol^−1^), F3MEE (18.7 kJ mol^−1^), and F6DEE (41.5 kJ mol^−1^) (**Figure** **3**).

As shown in the charge‒discharge voltage curves of the graphite|Li cells, voltage plateaus were observed in the range of 0.5–1.3 V in strongly solvating DME with low *µ*_Li_^+^ (0 kJ mol^−1^). This reaction corresponds to Li^+^–solvent cointercalation forming Li–solvent–graphite intercalation compounds (GICs) with an interlayer distance of 1.11 nm,^[^ ^34^ ^]^ which is remarkably expanded from the value of 0.335 nm for pure graphite, as confirmed by X‐ray diffraction (XRD) patterns (Figure S5, Supporting Information). In contrast, in weakly solvating F6DEE with high *µ*_Li_^+^ (41.5 kJ mol^−1^), voltage plateaus were observed in the range of 0–0.25 V, with a reversible capacity of over 350 mAh g^−1^. This reaction corresponds to Li^+^ intercalation forming Li‐GIC LiC_6_ (theoretical capacity: 372 mAh g^−1^) with a less expanded interlayer distance of 0.37 nm^[^ ^12^ ^]^ (Figure S5, Supporting Information). In moderately solvating F3MEE with intermediate *µ*_Li_^+^ (18.7 kJ mol^−1^), we observed Li^+^ intercalation, but the reversible capacity was much lower because of the incomplete inhibition of Li^+^–solvent cointercalation. Hence, unfavorable Li^+^–solvent cointercalation could be suppressed when the electrolyte has certain high *µ*_Li_^+^.

In addition, we studied the reactions of graphite in various solvents over a wide range of *µ*_Li_^+^ values (**Figure** **4**a; Figures S6–S17, Supporting Information). We found a threshold of *µ*_Li_^+^ at 10–25 kJ mol^−1^ that dominantly determined whether Li^+^ intercalation or Li^+^–solvent cointercalation occurred.

**Figure 4:**
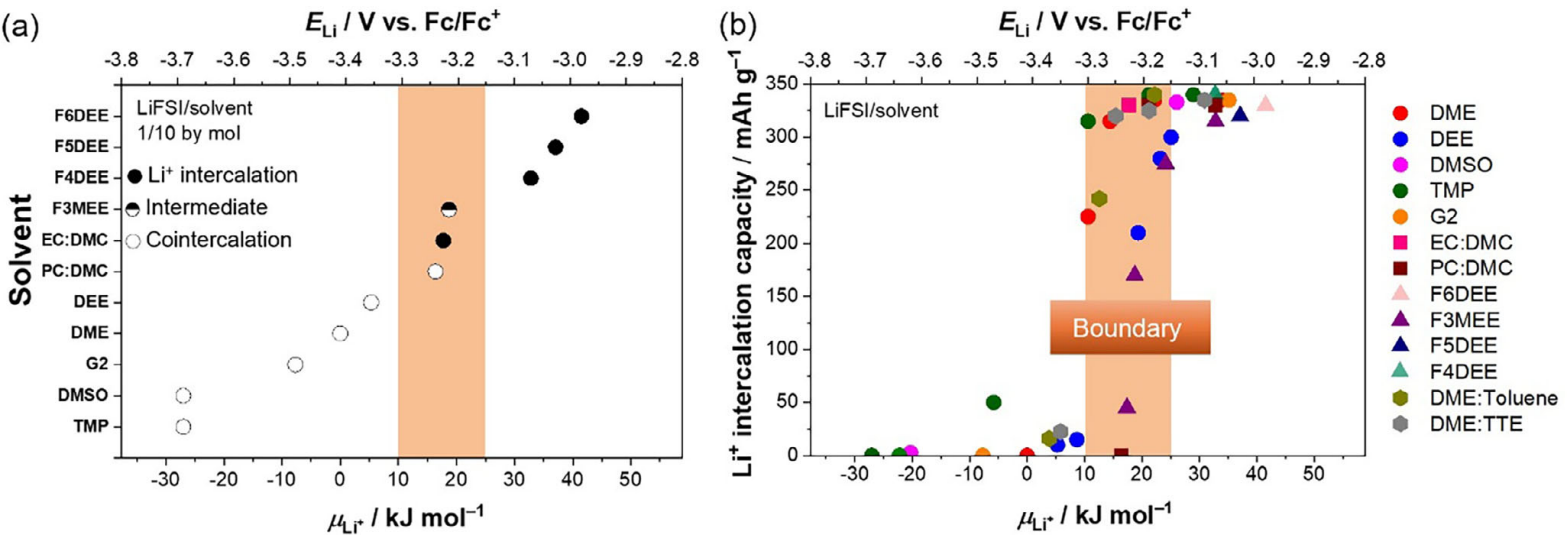
a) Li^+^ intercalation behavior of graphite electrodes in LiFSI/solvent (1/10 by mol) electrolytes with diverse solvents and b) reversible Li^+^ intercalation capacities of graphite|Li half cells in a voltage range of 0–0.25 V in diverse electrolytes depending on the Li metal electrode potential *E*_Li_ and electrolyte Li^+^ chemical potentials *µ*_Li_^+^ (vs LiFSI/DME, 1/10).

Having discovered threshold *µ*_Li_^+^ for different solvation abilities, we next studied LiFSI/DEE electrolytes at different salt concentrations (Figure 3; Figure S7, Supporting Information). *µ*_Li_^+^ increased with increasing salt concentration (Table S2, Supporting Information). As shown in Figure 3, in dilute LiFSI/DEE (1/10 by mol) with low *µ*_Li_^+^ (5.31 kJ mol^−1^), Li^+^–solvent cointercalation occurred at 0.5–1.3 V. At an intermediate concentration (1/1.8 by mol) with intermediate *µ*_Li_^+^ (19.3 kJ mol^−1^), we observed both Li^+^ intercalation (0–0.25 V) and Li^+^–solvent cointercalation (sloping curve in 0.5–1.3 V). In contrast, at a high concentration (1/1.5 by mol) with high *µ*_Li_^+^ (25.1 kJ mol^−1^), we observed Li^+^ intercalation with a reversible capacity of over 350 mAh g^−1^. These different reactions are proven by the XRD patterns (Figure S18, Supporting Information) and the colors (Figure S19, Supporting Information) of the graphite electrodes. Formation of different GICs was also demonstrated for dilute to concentrated LiFSI/DME (Figures S20 and S21, Supporting Information). Notably, the threshold *µ*_Li_^+^ that dominates the graphite reactions in LiFSI/DEE and LiFSI/DME at various concentrations is 10–25 kJ mol^−1^, which is the same as that for different solvation abilities.

It should be noted that the graphite reactions cannot be defined by the salt concentration. For example, the threshold concentrations for reversible Li^+^ intercalation are remarkably different for LiFSI/DEE (1/1.6 by mol, Figure S7d, Supporting Information) and LiFSI/F3MEE (1/4.4 by mol, Figure S9c, Supporting Information), but their *µ*_Li_^+^ values are similar (23.2 and 24.1 kJ mol^−1^, respectively). This result indicates that *µ*_Li_^+^ can be a descriptor of graphite reactions. This *µ*_Li_^+^ threshold can also be applied to localized highly concentrated electrolytes, LiFSI/DME:toluene and LiFSI/DME:1,1,2,2‐tetrafluoroethyl 2,2,3,3‐tetrafluoropropyl ether (TTE) (Figures S16 and S17, Supporting Information).

To generalize the relationship between *µ*_Li_^+^ and graphite negative electrode reactions, we studied 39 electrolyte formulations with different solvents and salt concentrations (Figures S6–S17, Supporting Information). To judge which reaction (Li^+^ intercalation or Li^+^–solvent cointercalation) predominantly occurred, we extracted the reversible capacities in a voltage range of 0–0.25 V for graphite|Li cells, which are fully attributed to Li^+^ intercalation. Figure 4b shows the relationship between *µ*_Li_^+^ and the Li^+^ intercalation capacity in various electrolytes. In low‐*µ*_Li_^+^ electrolytes (*µ*_Li_^+^ < 10 kJ mol^−1^), the Li^+^ intercalation capacities are generally small because of the predominant Li^+^–solvent cointercalation. In high‐*µ*_Li_^+^ electrolytes (*µ*_Li_^+^ > 25 kJ mol^−1^), the Li^+^ intercalation capacities are generally high (> 300 mAh g^−1^) because of suppressed Li^+^–solvent cointercalation. For the intermediate‐*µ*_Li_^+^ electrolytes (10 kJ mol^−1^ < *µ*_Li_^+^ < 25 kJ mol^−1^), the Li^+^ intercalation capacities significantly vary, suggesting that such *µ*_Li_^+^ values are in the boundary region. This result demonstrates that graphite negative electrode reactions are strongly correlated with the *µ*_Li_^+^ values in electrolytes.

### 2.3 Determining Factors for Graphite Negative Electrode Reactions

Finally, we discuss successful Li^+^ intercalation in high‐*µ*_Li_^+^ electrolytes. First, to judge whether this effect is kinetic or thermodynamic, we studied graphite reactions in boundary‐*µ_L_*_i_^+^ electrolytes (LiFSI/DME (1/4 and 1/3 by mol)) at various C‐rates from C/10 to 10C (Figure S22, Supporting Information). The solvent cointercalation over 0.5 V still occurred at higher C‐rates, indicating that the solvent cointercalation is not prevented in a kinetic way via an energy barrier. There would be non‐kinetic factors related to the thermodynamic quantity, *µ*_Li_^+^.

On this basis, we suppose that Li^+^ intercalation in high‐*µ*_Li_^+^ electrolytes is enabled by two mechanisms depending on *µ*_Li_^+^: i) formation of FSI anion‐derived SEI and ii) destabilization of solvated Li^+^. The SEI is known to be essential for graphite negative electrodes. High‐*µ*_Li_^+^ electrolytes commonly have extensive ion pairing (Figure 2; Figure S4, Supporting Information), which promotes an FSI anion‐derived SEI formation on graphite.^[^ ^13^ ^]^ Similar tendency was also reported for Li metal and Si‐based negative electrodes.^[^ ^35^, ^36^ ^]^ This unique SEI contributes, to some extent, to reversible Li^+^ intercalation by blocking solvated Li^+^ from intercalation. However, we found that the SEI chemistry did not remarkably change in the boundary *µ*_Li_^+^ region. As shown in Figure S23 (Supporting Information), both LiFSI/DME (1/2 and 1/3 by mol) resulted in similar FSI anion‐derived SEI but showed remarkably different graphite reactions. In addition, solvent cointercalation could not be suppressed in LiFSI/DME (1/3 by mol) even after forming SEI in LiFSI/DME (1/2 by mol) with higher *µ*_Li_^+^ (Figure S24, Supporting Information). Similarly, EC‐derived SEI was reported to be unable to inhibit solvent cointercalation.^[^ ^27^, ^37^ ^]^ On the other hand, solvent cointercalation was suppressed in salt‐concentrated propylene carbonate (PC) electrolytes (high *µ*_Li_^+^) with less SEI‐forming LiClO_4_ or LiPF_6_.^[^ ^38^ ^]^ Hence, although the SEI is essential for suppressing electrolyte decomposition, it may not be the only dominant factor. On the other hand, LiFSI/DME (1/2 and 1/3 by mol) electrolytes show a large difference of ≈10 kJ mol^−1^ in *µ*_Li_^+^, suggesting that the Li^+^ solvation is remarkably destabilized at higher concentration. We suppose that destabilized Li^+^ solvation (i.e., high *µ*_Li_^+^) also decreases the thermodynamic stability of resulting Li‐solvent‐GICs, which is responsible for suppressing solvent cointercalation. Previous reports show that the thermodynamic stability (i.e., formation potential) of Na‐solvent‐GICs can be tuned by the solvation structure of Na^+^.^[^ ^39^, ^40^ ^]^ A destabilized Na^+^ solvation (i.e., a weak solvation environment) leads to less shielding of the repulsion between the solvated Na^+^ in the interlayer of graphite, thus destabilizing the Na‐solvent‐GICs (its formation potential becomes lower).^[^ ^39^, ^40^ ^]^ Lower formation potential of Li‐solvent‐GICs was also observed in higher Li salt concentration electrolytes.^[^ ^29^ ^]^ On this basis, Li‐solvent‐GICs are generally destabilized in high‐*µ*_Li_^+^ electrolytes with destabilized Li^+^ solvation, which inhibits solvent cointercalation and instead favors Li^+^ intercalation after desolvation. Hence, the *µ*_Li_^+^ values in electrolytes strongly correlate with the thermodynamic reactivity of the graphite electrodes.

## 3 Conclusion

We discovered that *µ*_Li_^+^ is a quantitative descriptor of the Li^+^ intercalation behavior of graphite negative electrodes. Unfavorable Li^+^–solvent cointercalation is commonly suppressed when *µ*_Li_^+^ is increased (thus, Li^+^ is destabilized in the electrolyte) to above a certain threshold. This threshold is generally applicable to various types of LiFSI‐based electrolytes (e.g., highly concentrated electrolytes, localized highly concentrated electrolytes, and weakly coordinating electrolytes) to form inorganic‐rich SEI. On the basis of this insight, new electrolytes can be rationally designed, with a focus on *µ*_Li_^+^ to ensure the reversible reaction of graphite negative electrodes. The *µ*_Li_^+^ threshold may be shifted when the nature of SEI is significantly changed with SEI forming additives, extremely unstable solvent/salt for reduction, passivating binders, and functional surface coatings. High‐*µ*_Li_^+^ electrolytes upshift *E*_Li_ and thus are also promising for alleviating electrolyte reductive decomposition on Li metal and increasing its Coulombic efficiency.^[^ ^30^ ^]^ This work does not deny the contribution of FSI anion‐derived SEI to inhibiting solvent cointercalation but shows that destabilized Li^+^ solvation (higher *µ_L_*_i_^+^) is more important. We propose *µ*_Li_^+^ as a comprehensive descriptor including the contributions of both SEI and destabilized Li^+^ solvation to inhibiting solvent cointercalation. We recognize that the concept of Li bonds also well describes some battery reactions.^[^ ^41^ ^]^ The *µ*_Li_^+^ proposed here comprehensively describes the strength of Li bonds or the energetics of Li^+^ in a real solvation structure, thus further deepening the understanding of Li‐bond chemistry. A class of high‐*µ*_Li_^+^ electrolytes is worth exploring as alternatives for commercial EC electrolytes toward next‐generation LIBs with higher energy density and higher safety.

## 4 Experimental Section

### Materials

DME, DEE, dimethyl sulfoxide (DMSO), PC, dimethyl carbonate (DMC), trimethyl phosphate (TMP), EC:DMC (3:7 by volume), TTE solvents were purchased from Kishida Chemical Co. and G2 solvent was purchased from Kanto Chemical Co. as battery grade. Super dehydrated toluene was purchased from Fujifilm Wako Pure Chemical Corp. F6DEE, F5DEE, 1,2‐bis(2,2‐difluoroehoxy) ethane (F4DEE), F3MEE solvents (Daikin Industries, Ltd) and LiFSI salt (Nippon Shokubai Co., Ltd) were provided. All the chemicals were used without further purifying treatments. The LiFSI salt was dissolved in electrolyte solvents to prepare electrolyte solutions in an Ar‐filled glove box. The electrolyte containing 1 mmol dm^−3^ (mm) Fc (Sigma–Aldrich) was also prepared for measuring *E*_Li_ with reference to Fc/Fc^+^. Natural graphite powders were provided by SEC Carbon, Ltd. For fabricating graphite composite electrodes, the natural graphite powders and 12 wt.% polyvinylidene difluoride (PVdF, Kureha Corp.) solution were mixed in N‐methylpyrrolidone (NMP, Nacalai Tesque, Inc.). The slurry was uniformly coated on a copper current collector (18 µm thickness) with a coating thickness of 100 µm, and further vacuum‐dried at 120 °C. The resulting electrode sheets were punched out for obtaining disk electrodes with the size of 15.95 mmϕ.

### Evaluation of *µ*_Li_^+^

To quantify *µ*_Li_^+^ in an electrolyte, *E*_Li_ was measured with reference to the redox potential of Fc/Fc^+^. The electrochemical cell used in this experiment was composed of three electrodes (Figure S2g, Supporting Information): a Pt plate working electrode (Nilaco Corp.) and Li metal reference and counter electrodes (Honjo Metal Co., Ltd.) with electrolytes containing 1 mm Fc. Cyclic voltammetry was conducted using a Solartron 1470E+1400 (Solartron Analytical) to measure the redox potential of Fc/Fc^+^ with reference to Li reference electrode, from the midpoint of the oxidation/reduction peaks. Here, supposing that the redox potential of Fc/Fc^+^ was constant,^[^ ^31^ ^]^ *E*_Li_ was defined as Li electrode potential with reference to the Fc/Fc^+^ redox potential. As shown in Note S1 (Supporting Information), *µ*_Li_^+^ in an electrolyte can be derived from the *E*_Li_ evaluated in the same electrolyte.

(2) $$\mu_{Li^{+}}=FE_{\mathrm{Li}}+\mathrm{constant}$$

Here, *µ*_Li_^+^ was defined to be zero in a standard electrolyte, LiFSI/DME (1/10 by mol), and *µ*_Li_^+^ in various electrolytes was obtained as relative values with reference to that in LiFSI/DME (1/10 by mol).

### Charge–Discharge Measurements

The charge–discharge performance of natural graphite electrodes was evaluated at 25 °C with a 2032‐type coin cell employing a Li metal counter electrode and a glass fiber separator (GC‐50, Advantec). The coin cell parts from Hohsen Corp. and a charge–discharge unit (TOSCAT) from Toyo System Co., Ltd., were used. Charge–discharge voltage range was set at 0.01–2.5 V. Charge–discharge current of the ones corresponding to the values between C/100 and 10C was applied without using a constant‐voltage mode. *x*C rate was decided based on the current of 372*x* mA g^−1^ for the weight of natural graphite. All the electrochemical measurements were conducted under an Ar atmosphere. Charge–discharge measurements with electrolyte replacement were conducted by disassembling the graphite|Li coin cells with LiFSI/DME (1/2 by mol) after 3 cycle charge–discharge at C/10, carefully washing graphite electrodes using DME, reassembling the graphite|Li coin cells with washed graphite electrodes and LiFSI/DME (1/3 by mol) and finally being subjected to 3 cycle charge–discharge again.

### Materials Characterization

Raman spectroscopy (NRS‐5100 spectrometer, JASCO) with a laser excitation wavelength of 532 nm was used for analyzing the liquid structure of the electrolytes. The resolution of the spectrometer was 0.8 cm^−1^. The electrolytes were introduced into quartz cells with sealing in an Ar‐filled glove box for avoiding the contact with air, and the electrolytes were exposed to the laser through a quartz window. The measured Raman shifts were calibrated with a standard ethanol peak. To confirm the structural changes of the graphite electrodes after discharging, XRD was carried out ex situ under an Ar atmosphere with a SmartLab (RIGAKU) powder diffractometer (CuKα radiation). The surface composition of graphite electrodes after charge–discharge measurements was investigated using X‐ray photoelectron spectroscopy (JPS‐9010MC, JEOL) with Al Kα X‐ray source. Graphite samples were rinsed with electrolyte solvents three times in an Ar‐filled glove box. Thereafter, the samples were transferred into the chamber using a transfer vessel without exposure to air.

### Theoretical Calculations

The geometries of solvents, Li^+^, and solvated Li^+^ for the ground states were optimized at the B3LYP/6–311G+ (*d*, *p*) level using density functional theory (DFT) calculation. The energy of molecules was calculated at the B3LYP/6–311G+ (*d*, *p*) level. Gaussian16 at the University of Osaka was used for the DFT calculations.

## Conflict of Interest

The authors declare no conflict of interest.

## Author Contributions

Y. Kondo and H.N. contributed equally to this work. Y.Y. conceived and supervised the project. Y. Kondo and Y.Y. proposed the concept. Y. Kondo, H.N., Y. Katayama, and Y.Y. designed the experiments. Y. Kondo and H.N. conducted the experiments and the theoretical calculations. N.K., S.O., A.T., and S.Y. synthesized weakly coordinating fluorinated electrolyte solvents. All authors contributed to the discussion. Y. Kondo, H.N., and Y.Y. wrote the manuscript with the input from all authors.

## Supporting information



Supporting Information

## Data Availability

The data that support the findings of this study are available from the corresponding author upon reasonable request.
