# Supplementary material for: Electrolyte Li+ Chemical Potential Correlates with Graphite Negative Electrode Reactions in Lithium‐Ion Batteries
Source: Adv Mater. 2025 Oct 25;38(5):e14060. doi: 10.1002/adma.202514060 (PMC12822524; doi:10.1002/adma.202514060)
Supplement: Supplementary file 1 — Supporting Information [file ADMA-38-e14060-s001.pdf]

ADVANCED MATERIALS

Supporting Information

for *Adv. Mater.*, DOI 10.1002/adma.202514060

Electrolyte Li^+ Chemical Potential Correlates with Graphite Negative Electrode Reactions in Lithium-Ion Batteries

*Yasuyuki Kondo, Haruna Nakajima, Yu Katayama, Nao Kobayashi, Shinya Otani, Akinori Tani, Shigeaki Yamazaki and Yuki Yamada**

Supporting Information for

Electrolyte Li^+ Chemical Potential Correlates with Graphite Negative Electrode Reactions in Lithium-Ion Batteries

Yasuyuki Kondo, [†] *Haruna Nakajima,* [†] *Yu Katayama,* [†] *Nao Kobayashi,* [‡] *Shinya Otani,* [‡] *Akinori Tani,* [‡] *Shigeaki Yamazaki,* [‡] *Yuki Yamada* [*,†]

[†]SANKEN, The University of Osaka, Ibaraki, Osaka, 567-0047, Japan

[‡]Chemicals Division, Daikin Industries, Ltd., Settsu, Osaka, 566-8585, Japan

Supporting Note 1

To quantitively determine Li^+ chemical potential (μ_{Li+}) in an electrolyte, the Li electrode potential (E_{Li}) was evaluated as an electromotive force between Pt electrode and Li reference electrode in the cells shown below. Since the surface of the Li metal is covered with SEI, there are six phases as follows.

T (I) | Pt (II) | Fc, Fc^+, Li^+ (III) | Li^+, SEI (IV) | Li (V) | T (I')

T (I) and T (I') are the same metal and connected to a potentiometer. The E_{Li}, the electromotive force, is evaluated as the potential difference between T (I') and T (I).

$$E_{\mathrm{Li}} = \phi^{\mathrm{I}'} - \phi^{\mathrm{I}} \quad \text{[S1]}$$

Here ϕ denotes the inner potential of each phase superscripted. At equilibrium, the electrochemical potentials ($\tilde{\mu}$) of relevant reaction species are equal between phase II and III, phase III and IV, and phase IV and V as follows.

$$\tilde{\mu}_{\mathrm{Fc}}^{\mathrm{III}} = \tilde{\mu}_{\mathrm{Fc}^+}^{\mathrm{III}} + \tilde{\mu}_{\mathrm{e}}^{\mathrm{II}} \quad \text{[S2]}$$

$$\tilde{\mu}_{\mathrm{Li}^+}^{\mathrm{III}} = \tilde{\mu}_{\mathrm{Li}^+}^{\mathrm{IV}} \quad \text{[S3]}$$

$$\tilde{\mu}_{\mathrm{Li}^+}^{\mathrm{IV}} + \tilde{\mu}_{\mathrm{e}}^{\mathrm{V}} = \tilde{\mu}_{\mathrm{Li}}^{\mathrm{V}} \quad \text{[S4]}$$

Since the electrochemical potential of electron is equal between contacted metal phases ($\tilde{\mu}_{\mathrm{e}}^{\mathrm{II}} = \tilde{\mu}_{\mathrm{e}}^{\mathrm{I}}$, $\tilde{\mu}_{\mathrm{e}}^{\mathrm{V}} = \tilde{\mu}_{\mathrm{e}}^{\mathrm{I}'}$), equations [S2] and [S4] can be transformed as follows.

$$\tilde{\mu}_{\mathrm{Fc}}^{\mathrm{III}} = \tilde{\mu}_{\mathrm{Fc}^+}^{\mathrm{III}} + \tilde{\mu}_{\mathrm{e}}^{\mathrm{I}} \quad \text{[S5]}$$

$$\tilde{\mu}_{\mathrm{Li}^+}^{\mathrm{IV}} + \tilde{\mu}_{\mathrm{e}}^{\mathrm{I}'} = \tilde{\mu}_{\mathrm{Li}}^{\mathrm{V}} \quad \text{[S6]}$$

Combining equations [S5] and [S6] derives the following equation.

$$\tilde{\mu}_{\mathrm{e}}^{\mathrm{I}'} - \tilde{\mu}_{\mathrm{e}}^{\mathrm{I}} = \left(\tilde{\mu}_{\mathrm{Li}}^{\mathrm{V}} - \tilde{\mu}_{\mathrm{Li}^+}^{\mathrm{IV}}\right) - \left(\tilde{\mu}_{\mathrm{Fc}}^{\mathrm{III}} - \tilde{\mu}_{\mathrm{Fc}^+}^{\mathrm{III}}\right) \quad \text{[S7]}$$

The left side of equation [S7] can be expanded using chemical potentials (μ), Faradaic constant (F), and ϕ, as follows.

$$\tilde{\mu}_{\mathrm{e}}^{\mathrm{I}'} - \tilde{\mu}_{\mathrm{e}}^{\mathrm{I}} = \left(\mu_{\mathrm{e}}^{\mathrm{I}'} - F\phi^{\mathrm{I}'}\right) - \left(\mu_{\mathrm{e}}^{\mathrm{I}} - F\phi^{\mathrm{I}}\right) = -F\left(\phi^{\mathrm{I}'} - \phi^{\mathrm{I}}\right) = -FE_{\mathrm{Li}} \quad \text{[S8]}$$

Here we used $\mu_{\mathrm{e}}^{\mathrm{I}'} = \mu_{\mathrm{e}}^{\mathrm{I}}$, which holds because the phases I' and I are the same metal and thus the chemical potentials of electron therein are the same. Combining equations [S7] and [S8] derives the following equation regarding E_{Li}.

$$-FE_{\mathrm{Li}} = \left(\tilde{\mu}_{\mathrm{Li}}^{\mathrm{V}} - \tilde{\mu}_{\mathrm{Li}^+}^{\mathrm{IV}}\right) - \left(\tilde{\mu}_{\mathrm{Fc}}^{\mathrm{III}} - \tilde{\mu}_{\mathrm{Fc}^+}^{\mathrm{III}}\right) \quad \text{[S9]}$$

Using equation [S3], equation [S9] can be transformed as follows.

$$-FE_{\mathrm{Li}} = \left(\tilde{\mu}_{\mathrm{Li}}^{\mathrm{V}} - \tilde{\mu}_{\mathrm{Li}^+}^{\mathrm{III}}\right) - \left(\tilde{\mu}_{\mathrm{Fc}}^{\mathrm{III}} - \tilde{\mu}_{\mathrm{Fc}^+}^{\mathrm{III}}\right) \qquad \text{[S10]}$$

Then, this equation can be expanded using μ, F, and ϕ.

$$\begin{aligned} -FE_{\mathrm{Li}} &= \left(\mu_{\mathrm{Li}}^{\mathrm{V}} - \mu_{\mathrm{Li}^+}^{\mathrm{III}} - F\phi^{\mathrm{III}}\right) - \left(\mu_{\mathrm{Fc}}^{\mathrm{III}} - \mu_{\mathrm{Fc}^+}^{\mathrm{III}} - F\phi^{\mathrm{III}}\right) \\ &= \left(\mu_{\mathrm{Li}}^{\mathrm{V}} - \mu_{\mathrm{Li}^+}^{\mathrm{III}}\right) - \left(\mu_{\mathrm{Fc}}^{\mathrm{III}} - \mu_{\mathrm{Fc}^+}^{\mathrm{III}}\right) \qquad \text{[S11]} \end{aligned}$$

In equation [S11], $\mu_{\mathrm{Li}}^{\mathrm{V}}$ (chemical potential of Li in Li metal phase) is constant in our experiment of using various electrolytes. Besides, we suppose that the redox potential of $\mathrm{Fc/Fc^+}$ is constant, which corresponds to the constant value of $\left(\mu_{\mathrm{Fc}}^{\mathrm{III}} - \mu_{\mathrm{Fc}^+}^{\mathrm{III}}\right)$. As a result, equation [S11] can be simplified as follows.

$$-FE_{\mathrm{Li}} = -\mu_{\mathrm{Li}^+}^{\mathrm{III}} + \mathrm{constant} \qquad \text{[S12]}$$

Hence, the following formula in the main text holds.

$$\mu_{\mathrm{Li}^+}^{\mathrm{III}} = FE_{\mathrm{Li}} + \mathrm{constant} \qquad \text{[S13]}$$

Table S1. Data list of various LiFSI/solvent (1/10 by mol) electrolytes. Li electrode potentials (E_{Li}), electrolyte Li^+ chemical potentials (μ_{Li^+}) with reference to that in LiFSI/DME (1/10 by mol), reversible capacities in a voltage range of 0-0.25 V vs. Li at 1st and 5th cycles, and classification of formed graphite intercalation compounds are shown. For classification, Li^+ intercalation forms "Li-GIC," while Li^+-solvent cointercalation forms "Li-solvent-GIC." "Incomplete Li-GIC" means that solvent cointercalation is not fully suppressed and thus Li+ intercalation occurs with less reversibility and/or lower capacity. "No GIC" means that neither Li^+ intercalation nor Li^+-solvent cointercalation occurs.

**Electrolyte (salt/solvent molar ratio)**	**E_{Li} (V vs. Fc/Fc^+)**	**μ_{Li^+} (kJ mol^{-1})**	**Reversible capacity at 1st cycle (mAh g^{-1})**	**Reversible capacity at 5th cycle (mAh g^{-1})**	**Classification A: Li-GIC B: Incomplete Li-GIC C: Li-solvent-GIC D: No GIC**
LiFSI/TMP (1/10)	-3.69	-27.0	0	0	D
LiFSI/DMSO (1/10)	-3.69	-27.0	0	0	D
LiFSI/G2 (1/10)	-3.49	-7.72	0	0	C
LiFSI/DME (1/10)	-3.41	0	0	0	C
LiFSI/DEE (1/10)	-3.355	5.31	10	5	C
LiFSI/PC:DMC (3:7, v/v) (1/10)	-3.24	16.4	0	0	D
LiFSI/EC:DMC (3:7, v/v) (1/10)	-3.226	17.4	330	330	A
LiFSI/F3MEE (1/10)	-3.216	18.7	170	170	B
LiFSI/F4DEE (1/10)	-3.07	32.8	340	340	A
LiFSI/F5DEE (1/10)	-3.026	37.1	320	325	A
LiFSI/F6DEE (1/10)	-2.98	41.5	330	335	A

Table S2. Data list of various LiFSI electrolytes with diverse solvents and salt concentrations. Li electrode potentials (E_{Li}), electrolyte Li^+ chemical potentials (μ_{Li^+}) with reference to that in LiFSI/DME (1/10 by mol), reversible capacities in a voltage range of 0-0.25 V vs. Li at 1st and 5th cycles, and classification of formed graphite intercalation compounds are shown. For classification, Li^+ intercalation forms "Li-GIC," while Li^+-solvent cointercalation forms "Li-solvent-GIC." "Incomplete Li-GIC" means that solvent cointercalation is not fully suppressed and thus Li+ intercalation occurs with less reversibility and/or lower capacity. "No GIC" means that neither Li^+ intercalation nor Li^+-solvent cointercalation occurs.

**Electrolyte (salt/solvent molar ratio)**	**E_{Li} (V vs. Fc/Fc^+)**	**μ_{Li^+} ($kJ\ mol^{-1}$)**	**Reversible capacity at 1st cycle ($mAh\ g^{-1}$)**	**Reversible capacity at 5th cycle ($mAh\ g^{-1}$)**	**Classification A: Li-GIC B: Incomplete Li-GIC C: Li-solvent-GIC D: No GIC**
LiFSI/DME (1/10)	-3.41	0	0	0	C
LiFSI/DME (1/3)	-3.3	10.6	225	130	C
LiFSI/DME (1/2.5)	-3.26	14.5	315	300	B
LiFSI/DME (1/2)	-3.18	22.2	335	335	A
LiFSI/DME (1/1.4)	-3.06	33.8	335	335	A
LiFSI/DEE (1/10)	-3.355	5.31	10	5	C
LiFSI/DEE (1/4.2)	-3.32	8.68	15	5	C
LiFSI/DEE (1/1.8)	-3.21	19.3	210	185	C
LiFSI/DEE (1/1.6)	-3.17	23.2	280	290	A
LiFSI/DEE (1/1.5)	-3.15	25.1	300	310	A
LiFSI/DMSO (1/10)	-3.69	-27.0	0	0	D
LiFSI/DMSO (1/6)	-3.62	-20.2	0	0	D
LiFSI/DMSO (1/2)	-3.14	26.1	325	325	A

LiFSI/PC:DMC (3:7, v/v) (1/10)	-3.24	16.4	0	0	D
LiFSI/PC:DMC (3:7, v/v) (1/6)	-3.19	21.2	330	330	A
LiFSI/PC:DMC (3:7, v/v) (1/3)	-3.07	32.8	330	340	A
LiFSI/TMP (1/10)	-3.69	-27.0	0	0	D
LiFSI/TMP (1/6)	-3.64	-22.2	0	0	D
LiFSI/TMP (1/4)	-3.47	-5.79	50	50	B
LiFSI/TMP (1/3)	-3.3	10.6	315	320	A
LiFSI/TMP (1/2.5)	-3.19	21.2	340	340	A
LiFSI/TMP (1/2)	-3.11	28.9	340	340	A
LiFSI/G2 (1/10)	-3.49	-7.72	0	0	C
LiFSI/G2 (1/1)	-3.046	35.1	333	335	A
LiFSI/F3MEE (1/20)	-3.23	17.4	45	45	C
LiFSI/F3MEE (1/10)	-3.216	18.7	170	170	B
LiFSI/F3MEE (1/4.4)	-3.16	24.1	280	275	A
LiFSI/F3MEE (1/2)	-3.07	32.8	315	325	A

Table S3. Data list of various localized high-concentration electrolytes composed of 1.5 M LiFSI and DME:toluene or DME:HFE at different mixing ratios. Li electrode potentials (E_{Li}), electrolyte Li^+ chemical potentials (μ_{Li^+}) with reference to that in LiFSI/DME (1/10 by mol), reversible capacities in a voltage range of 0-0.25 V vs. Li at 1st and 5th cycles, and classification of formed graphite intercalation compounds are shown. For classification, Li^+ intercalation forms "Li-GIC," while Li^+-solvent cointercalation forms "Li-solvent-GIC." "Incomplete Li-GIC" means that solvent cointercalation is not fully suppressed and thus Li+ intercalation occurs with less reversibility and/or lower capacity. "No GIC" means that neither Li^+ intercalation nor Li^+-solvent cointercalation occurs.

**Electrolyte (solvent/solvent molar ratio)**	**E_{Li} (V vs. Fc/Fc^+)**	**μ_{Li^+} (kJ mol^{-1})**	**Reversible capacity at 1st cycle (mAh g^{-1})**	**Reversible capacity at 5th cycle (mAh g^{-1})**	**Classification<br>A: Li-GIC<br>B: Incomplete Li-GIC<br>C: Li-solvent-GIC<br>D: No GIC**
1.5 M LiFSI/ DME:toluene (9/1)	-3.37	3.86	16	0	C
1.5 M LiFSI/ DME:toluene (5/5)	-3.28	12.5	240	120	C
1.5 M LiFSI/ DME:toluene (3.5/6.5)	-3.18	22.2	340	340	A
1.5 M LiFSI/ DME:TTE (9/1)	-3.35	5.79	20	10	C
1.5 M LiFSI/ DME:TTE (6/4)	-3.25	15.4	320	310	B
1.5 M LiFSI/ DME:TTE (5/5)	-3.19	21.2	325	330	A
1.5 M LiFSI/ DME:TTE (4/6)	-3.09	30.9	335	340	A

Table S4. Solvation energies (E_{sol}) calculated for the optimized structures of solvated Li^+ shown in Figure S5.

	DME	DEE	F3MEE	F6DEE
E_{sol} (kJ mol^{-1})	-459	-451	-412	-380

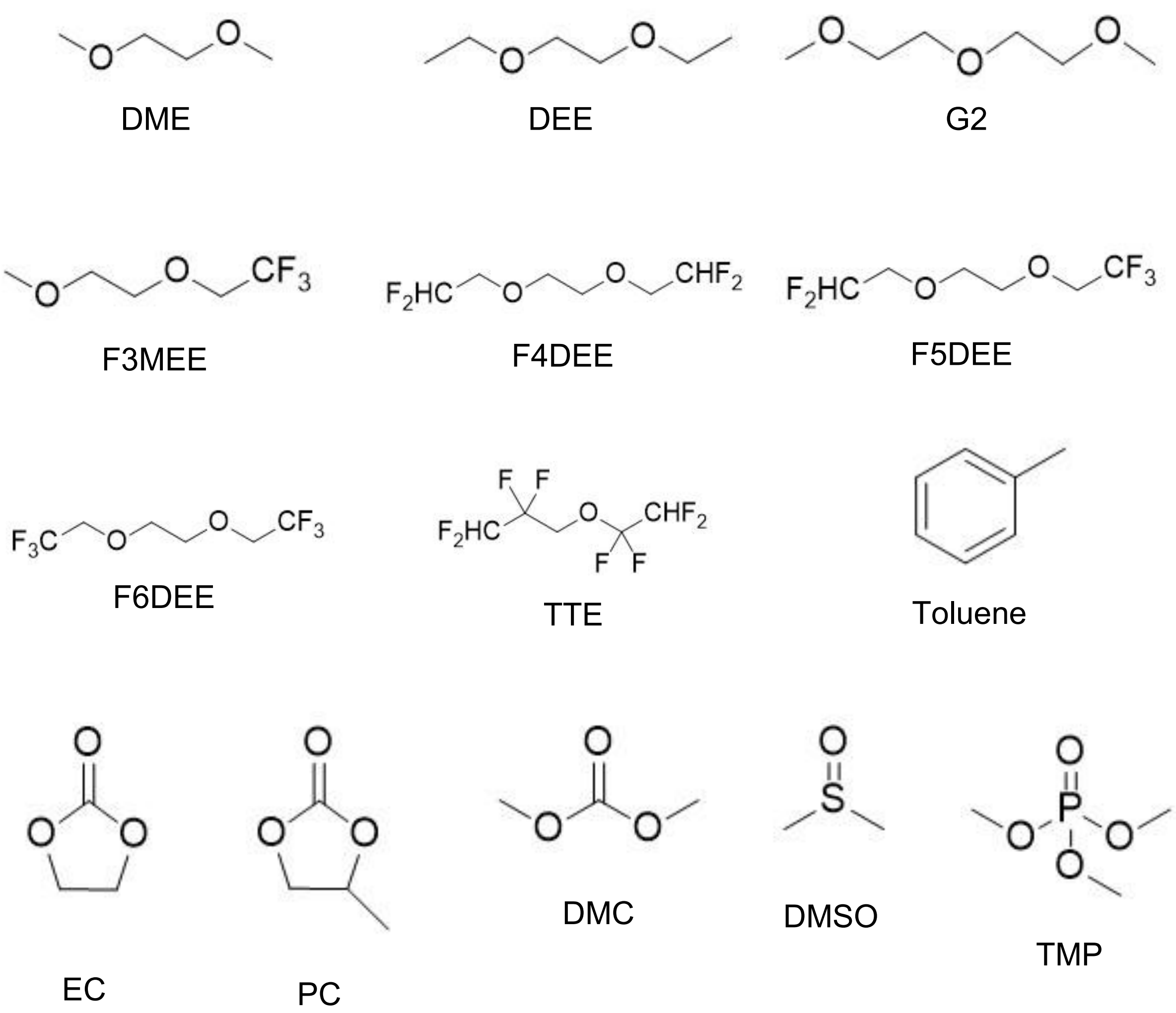

Figure S1. Molecular structures of the solvents used: DME, DEE, G2, F3MEE, F4DEE, F5DEE, F6DEE, TTE, Toluene, EC, PC, DMC, DMSO, and TMP.

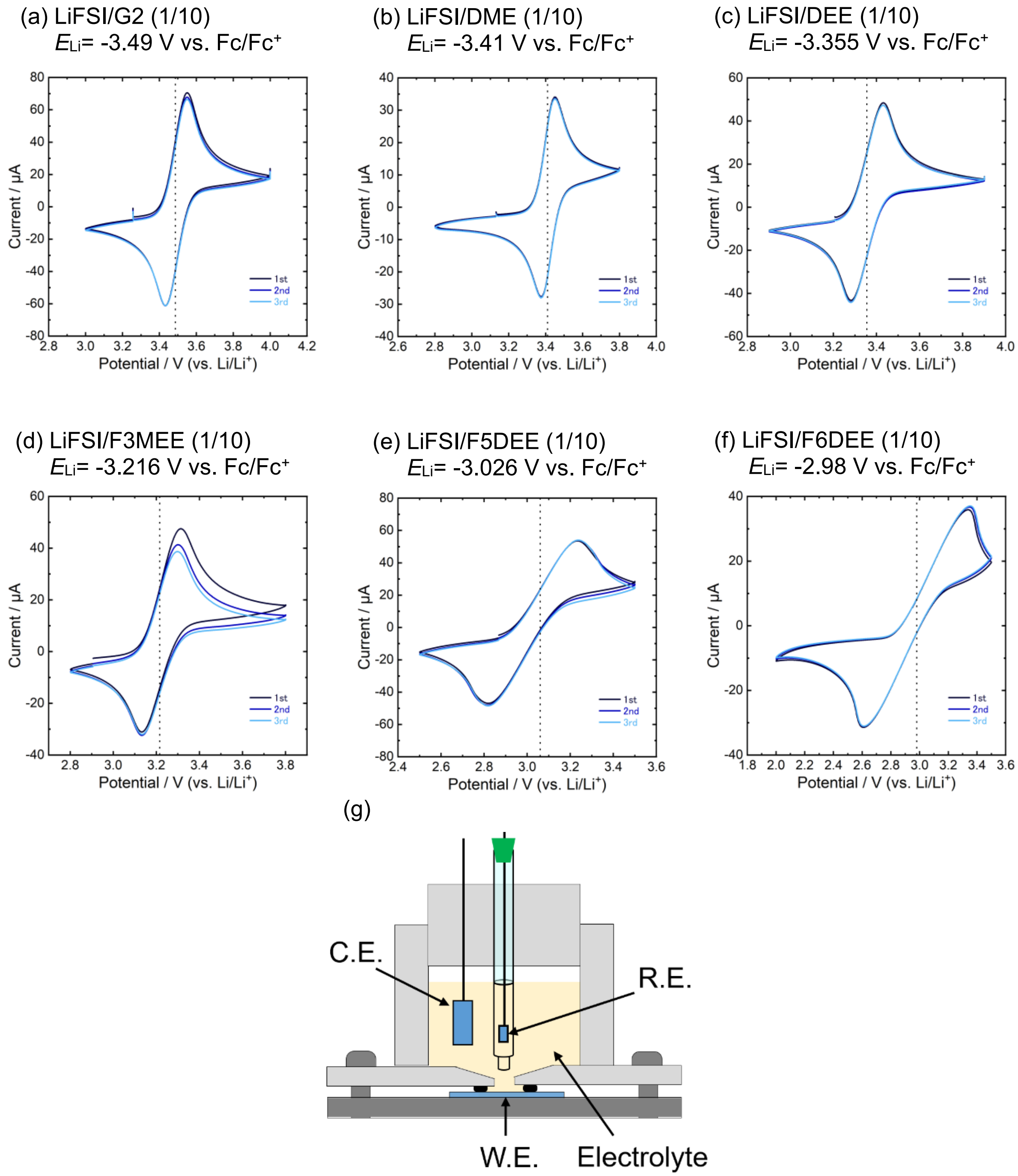

Figure S2. Cyclic voltammograms of 1 mM ferrocene (Fc) in (a) LiFSI/G2 (1/10 by mol), (b) LiFSI/DME (1/10 by mol), (c) LiFSI/DEE (1/10 by mol), (d) LiFSI/F3MEE (1/10 by mol), (e) LiFSI/F5DEE (1/10 by mol), and (f) LiFSI/F6DEE (1/10 by mol). (g) A schematic of three-electrode cell (W.E.: Pt, C.E.:Li, R.E.: Li) for cyclic voltammetry. The Li electrode potential (E_{Li}) was evaluated with reference to the redox potential of Fc/Fc^+.

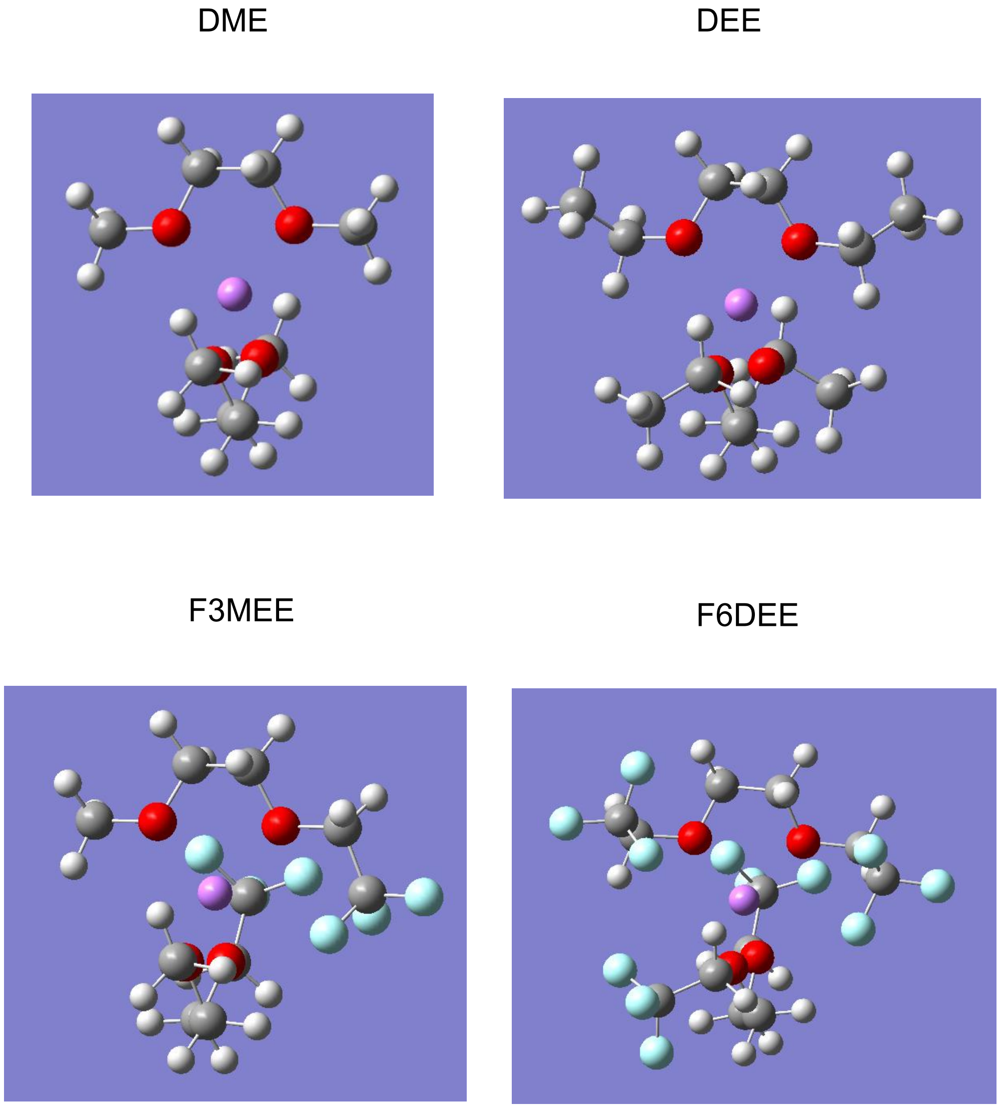

Figure S3. Optimized structures of lithium-ions solvated by 2 molecules of DME, DEE, F3MEE and F6DEE (Li: purple, C: gray, H: white, O: red and F: light blue).

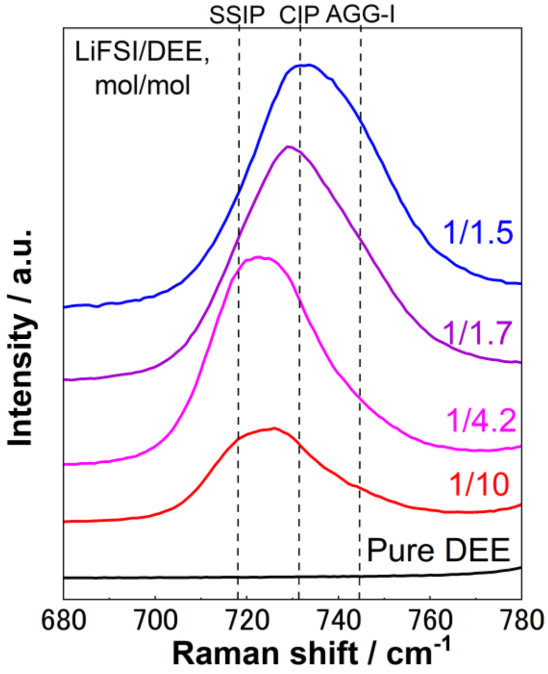

Figure S4. Raman spectra of LiFSI/DEE (1/10, 1/4.2, 1/1.7, 1/1.5 by mol) and pure DEE. The Raman band between 680 cm^{-1} and 780 cm^{-1} is derived from the vibration of FSI^-. The peak position represents the ion pairing states of Li^+ and FSI^-, namely solvent separate ion pair (SSIP), contact ion pair (CIP), and aggregate (AGG-I). The peak at higher wavenumber indicates more extensive ion pairs. Higher salt concentration leads to more extensive ion pairing.

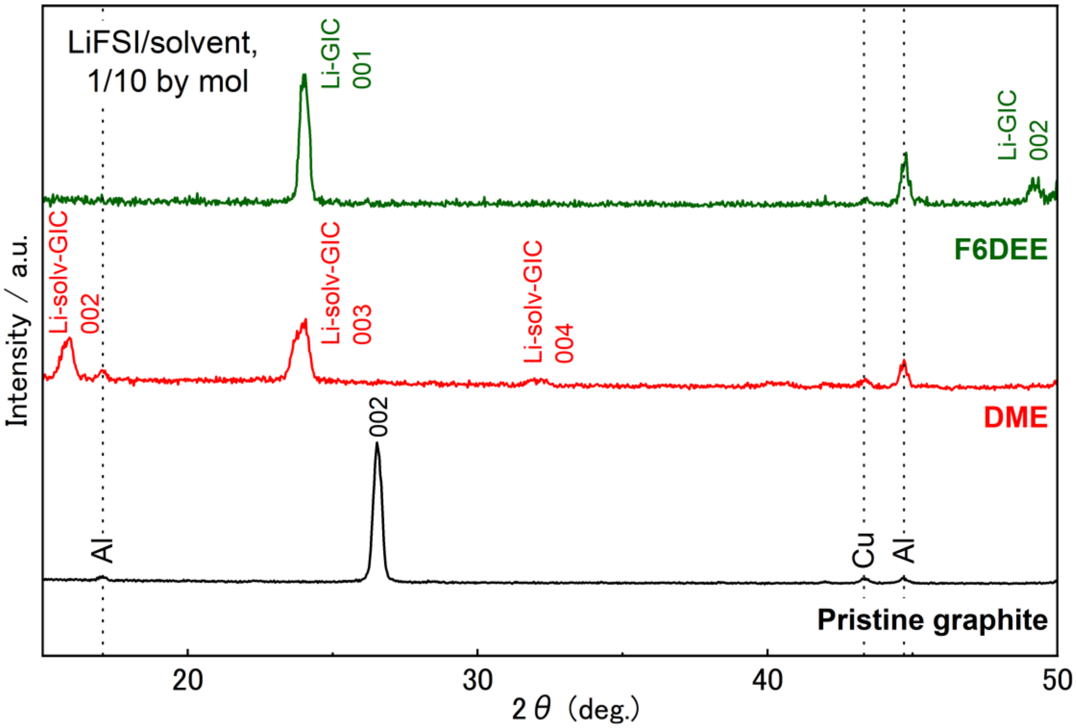

Li-GIC in LiFSI/F6DEE (1/10)

2θ	d / nm	00*l*	*Ic* / nm
24.04	0.370	001	0.370
49.18	0.185	002	0.370

Li-solvent-GIC in LiFSI/DME (1/10)

2θ	d / nm	00*l*	*Ic* / nm
15.94	0.556	002	1.11
24.04	0.370	003	1.11
32.22	0.278	004	1.11

Figure S5. XRD patterns of graphite electrodes discharged to around 0 V vs. Li metal in LiFSI/DME (1/10 by mol) and LiFSI/F6DEE (1/10 by mol), and pristine graphite electrode. The XRD pattern for the graphite in LiFSI/DME is indexed by an expanded interlayer distance of 1.11 nm as compared with 0.335 nm for pristine graphite, which suggests the formation of Li-solvent-GIC. On the other hand, the graphite in LiFSI/F6DEE is characterized by an interlayer distance of 0.37 nm, which indicates the formation of Li-GIC (LiC_6).

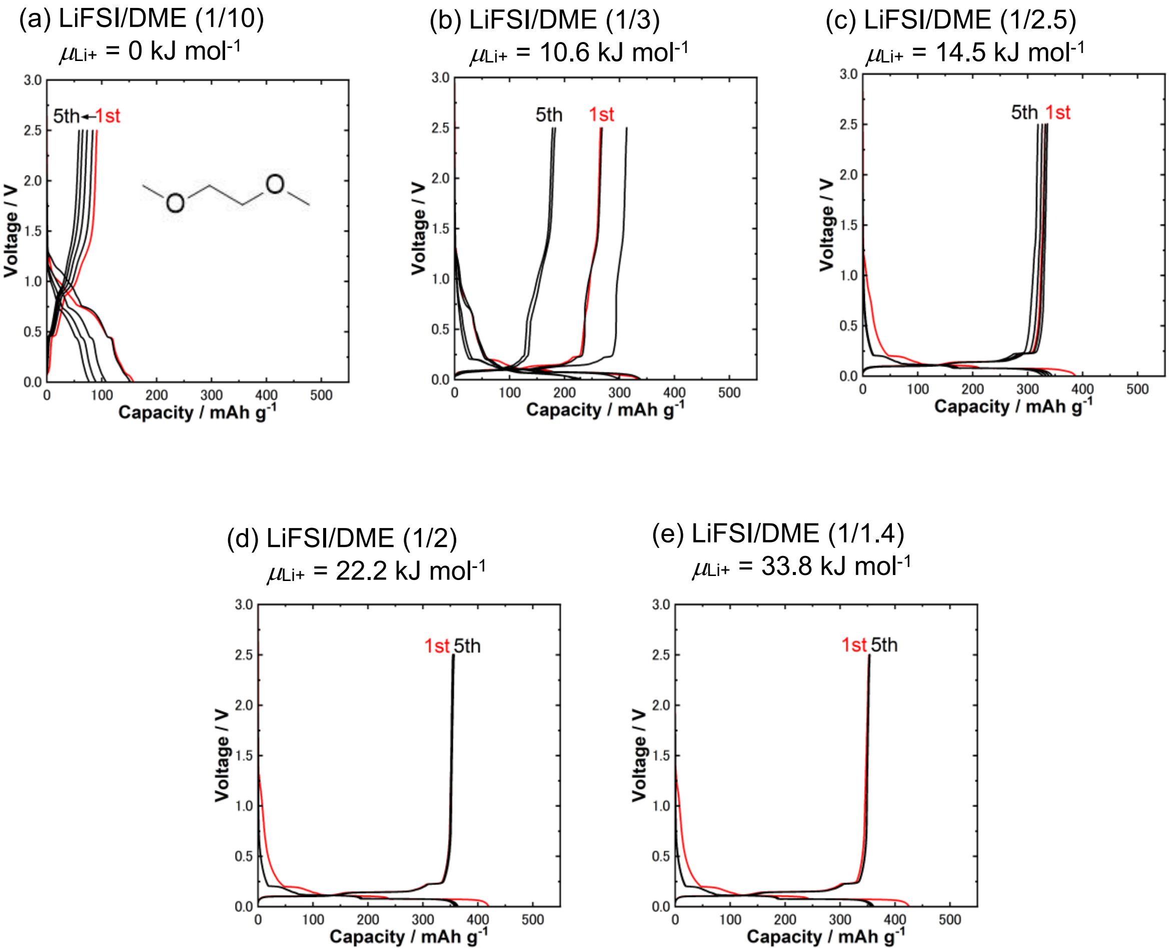

Figure S6. Charge-discharge curves of natural graphite/lithium cells with LiFSI/DME ((a) 1/10, (b) 1/3, (c) 1/2.5, (d) 1/2, and (e) 1/1.4 by mol) at C/10 rate (37.2 mA g^{-1}). The Li^+ chemical potential (μ_{Li+}) with reference to LiFSI/DME (1/10 by mol) is also given.

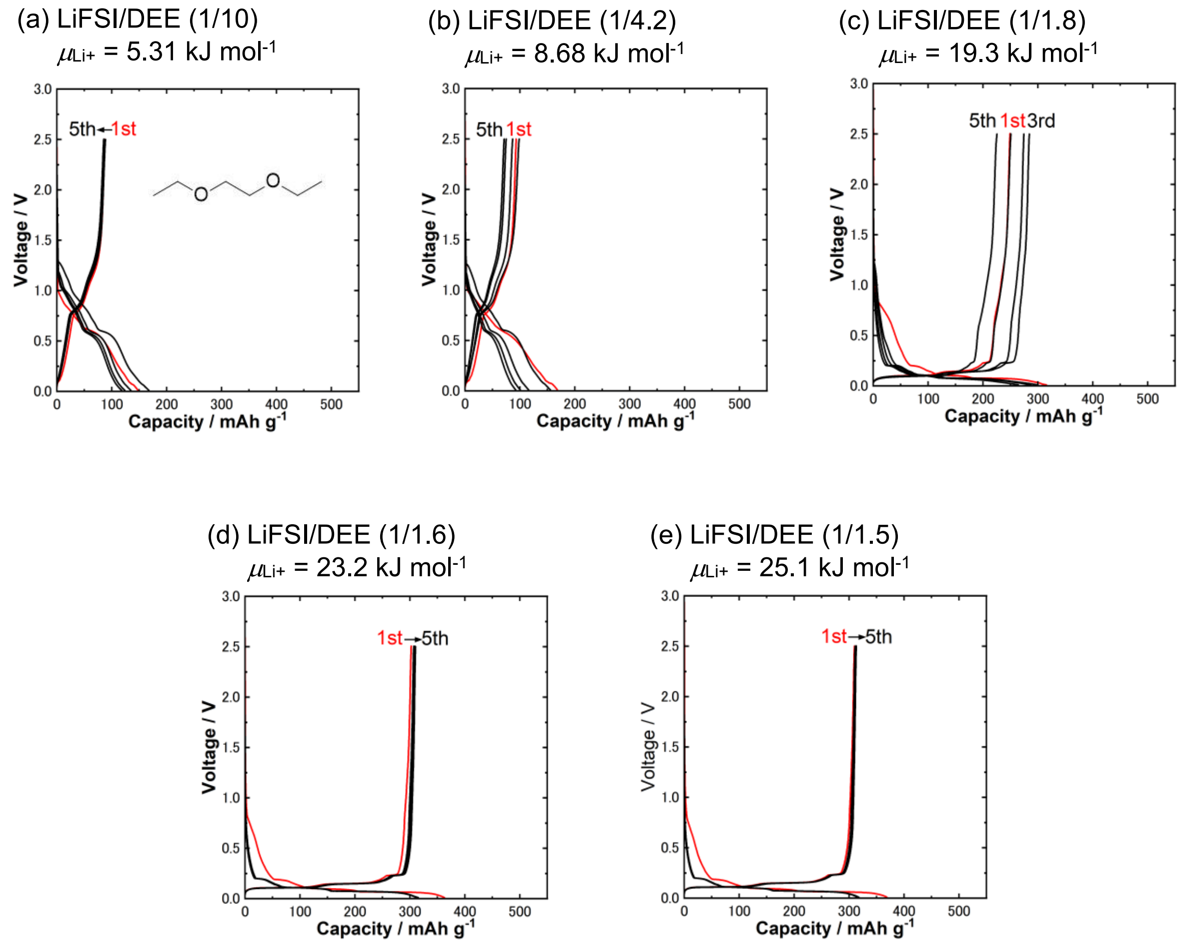

Figure S7. Charge-discharge curves of natural graphite/lithium cells with LiFSI/DEE ((a) 1/10, (b) 1/4.2, (c) 1/1.8, (d) 1/1.6, and (e) 1/1.5 by mol) at C/10 rate (37.2 mA g^{-1}). The Li^+ chemical potential (μ_{Li+}) with reference to LiFSI/DME (1/10 by mol) is also given.

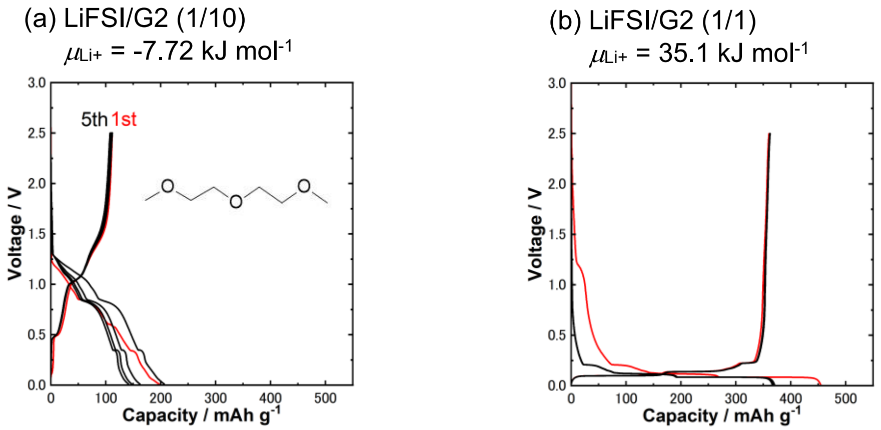

Figure S8. Charge-discharge curves of natural graphite/lithium cells with LiFSI/G2((a)1/10 and (b) 1/1 by mol). C rate was set at C/10 (37.2 mA g^{-1}) for LiFSI/G2 (1/10 by mol) and at C/100 (3.72 mA g^{-1}) for LiFSI/G2 (1/1 by mol). Lower C rate for the high concentration is due to lower ionic conductivity. The Li^+ chemical potential (μ_{Li+}) with reference to LiFSI/DME (1/10 by mol) is also given.

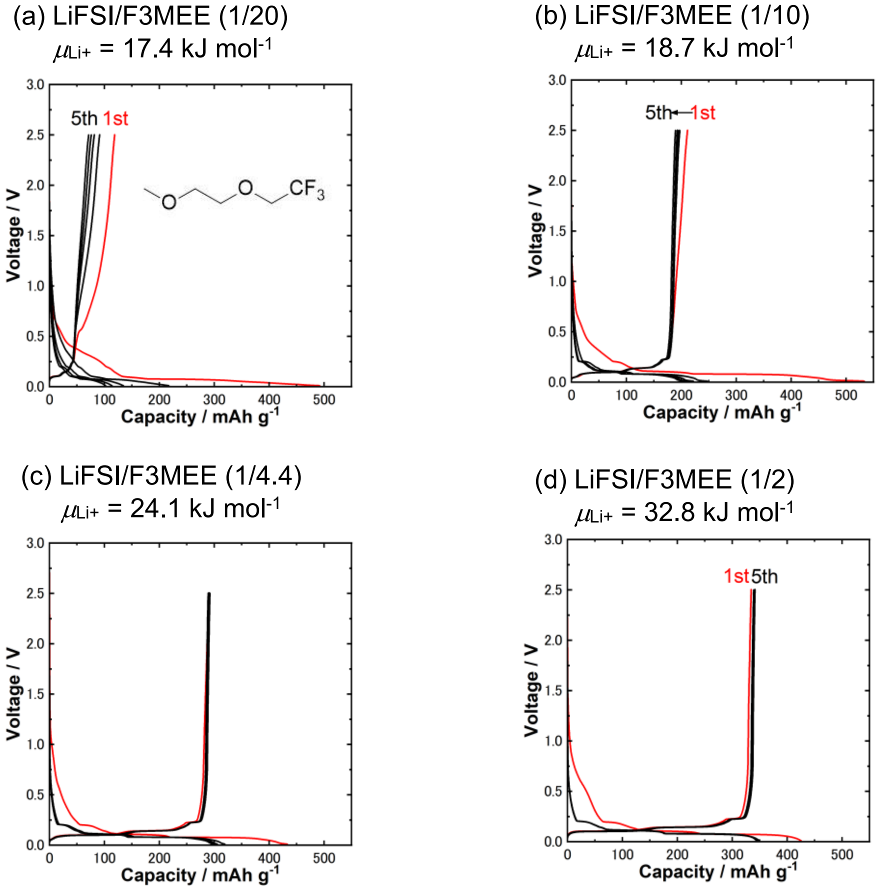

Figure S9. Charge-discharge curves of natural graphite/lithium cells with LiFSI/F3MEE ((a) 1/20, (b)1/10, (c)1/4.4, (d)1/2 by mol) at C/10 rate (37.2 mA g^{-1}). The Li^+ chemical potential (μ_{Li+}) with reference to LiFSI/DME (1/10 by mol) is also given.

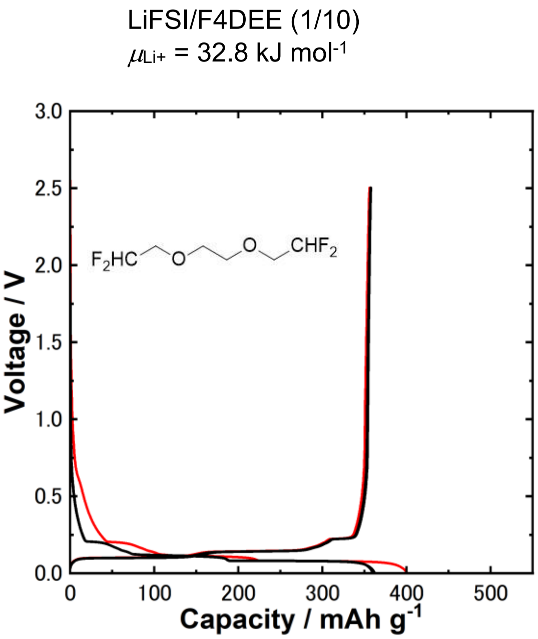

Figure S10. Charge-discharge curves of natural graphite/lithium cells with LiFSI/F4DEE (1/10 by mol) at C/10 rate (37.2 mA g^{-1}). The Li^+ chemical potential (μ_{Li+}) with reference to LiFSI/DME (1/10 by mol) is also given.

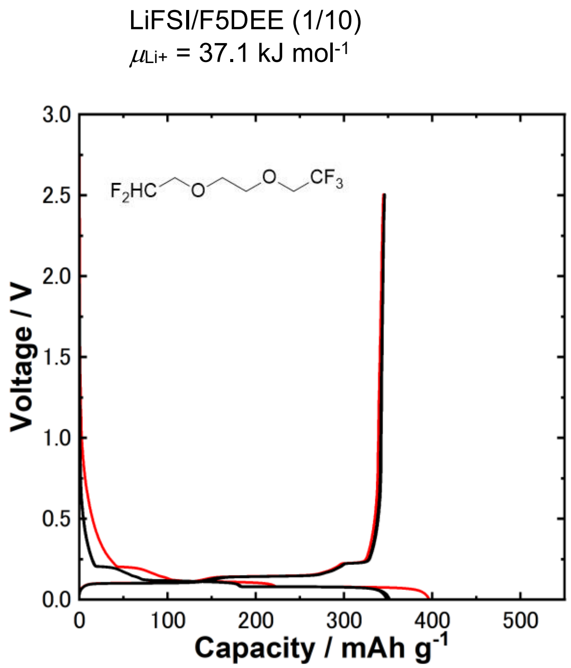

Figure S11. Charge-discharge curves of natural graphite/lithium cells with LiFSI/F5DEE (1/10 by mol) at C/10 rate (37.2 mA g^{-1}). The Li^+ chemical potential (μ_{Li+}) with reference to LiFSI/DME (1/10 by mol) is also given.

LiFSI/EC:DMC (3:7 by vol) (1/10)
μ_{Li+} = 17.4 kJ mol^{-1}

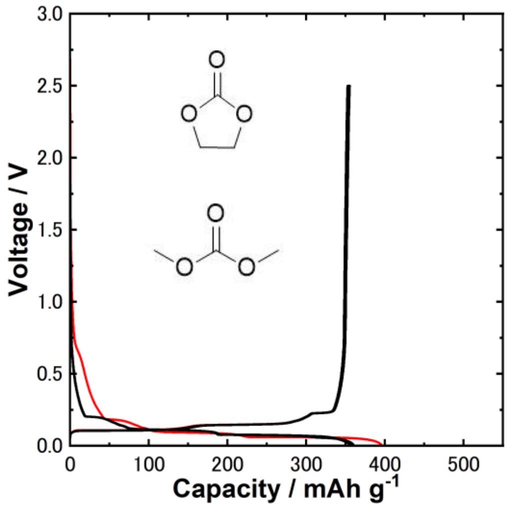

Figure S12. Charge-discharge curves of natural graphite/lithium cells with LiFSI/EC:DMC (3:7 by vol) (1/10 by mol) at C/10 rate (37.2 mAg^{-1}). The Li^+ chemical potential (μ_{Li+}) with reference to LiFSI/DME (1/10 by mol) is also given.

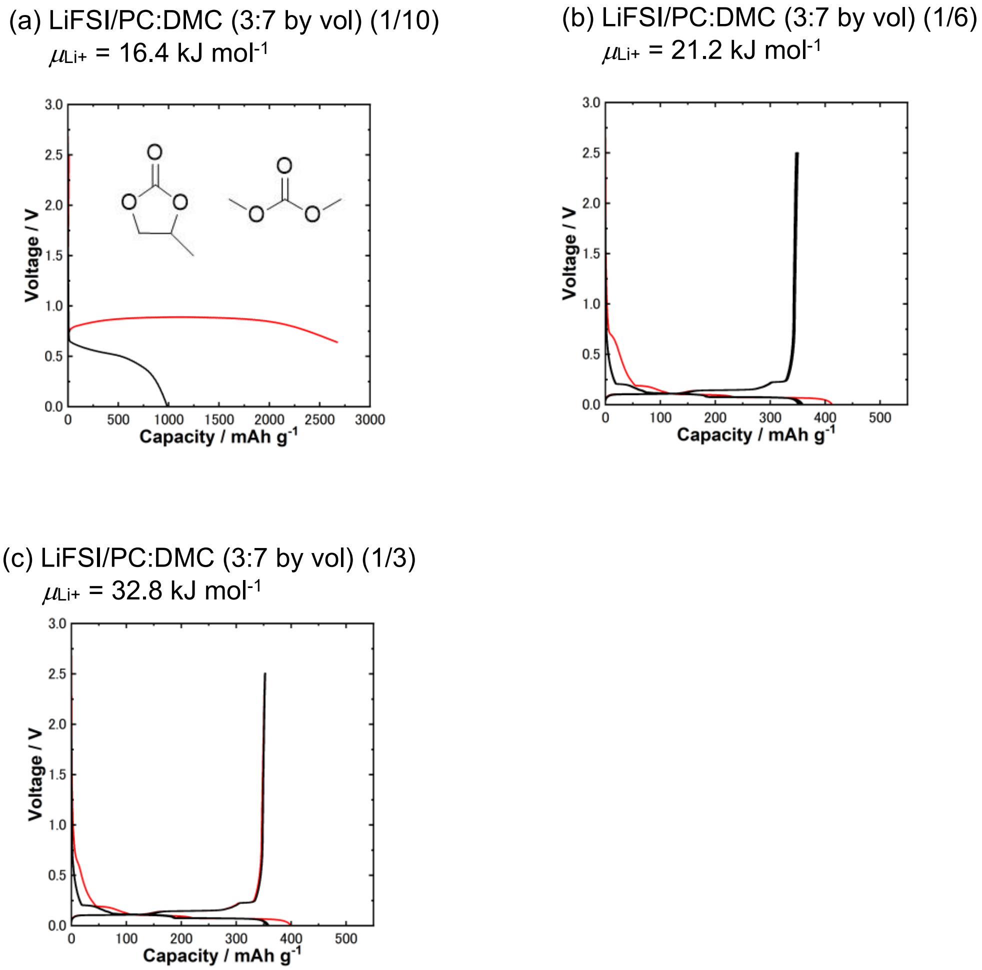

Figure S13. Charge-discharge curves of natural graphite/lithium cells with LiFSI/PC:DMC (3:7 by vol) ((a)1/10, (b)1/6, and (c) 1/3 by mol) at C/10 rate (37.2 mAg^{-1}). The Li^+ chemical potential (μ_{Li+}) with reference to LiFSI/DME (1/10 by mol) is also given.

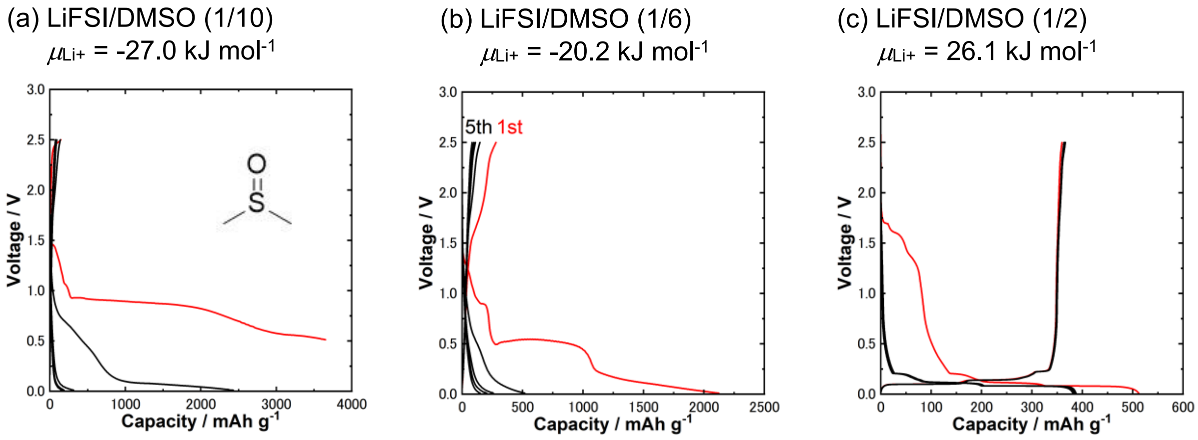

Figure S14. Charge-discharge curves of natural graphite/lithium cells with LiFSI/DMSO ((a) 1/10, (b)1/6, and (c) 1/2 by mol). C rate was set at C/10 (37.2 mA g^{-1}) for LiFSI/DMSO (1/10 and 1/6 by mol) and at C/100 (3.72 mA g^{-1}) for LiFSI/DMSO (1/2 by mol). Lower C rate for the high concentration is due to lower ionic conductivity. The Li^+ chemical potential (μ_{Li+}) with reference to LiFSI/DME (1/10 by mol) is also given.

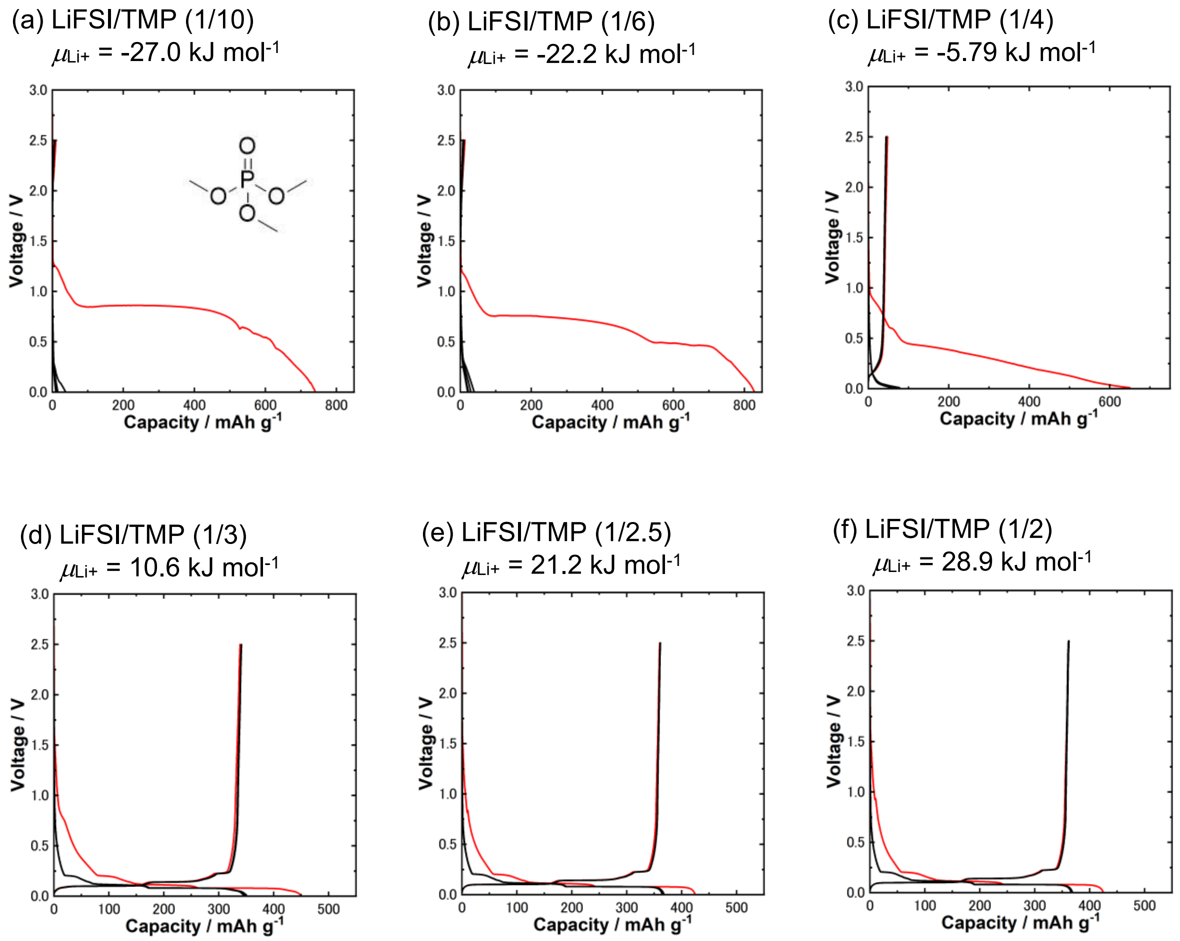

Figure S15. Charge-discharge curves of natural graphite/lithium cells with LiFSI/TMP ((a) 1/10, (b) 1/6, (c) 1/4, (d) 1/3, (e) 1/2.5, and (f) 1/2 by mol). C rate was set at C/10 (37.2 mA g^{-1}) for LiFSI/TMP (1/10, 1/6, and 1/4 by mol) and at C/100 (3.72 mA g^{-1}) for LiFSI/TMP (1/3, 1/2.5, and 1/2 by mol). Lower C rate for the high concentration is due to lower ionic conductivity. The Li^+ chemical potential (μ_{Li+}) with reference to LiFSI/DME (1/10 by mol) is also given.

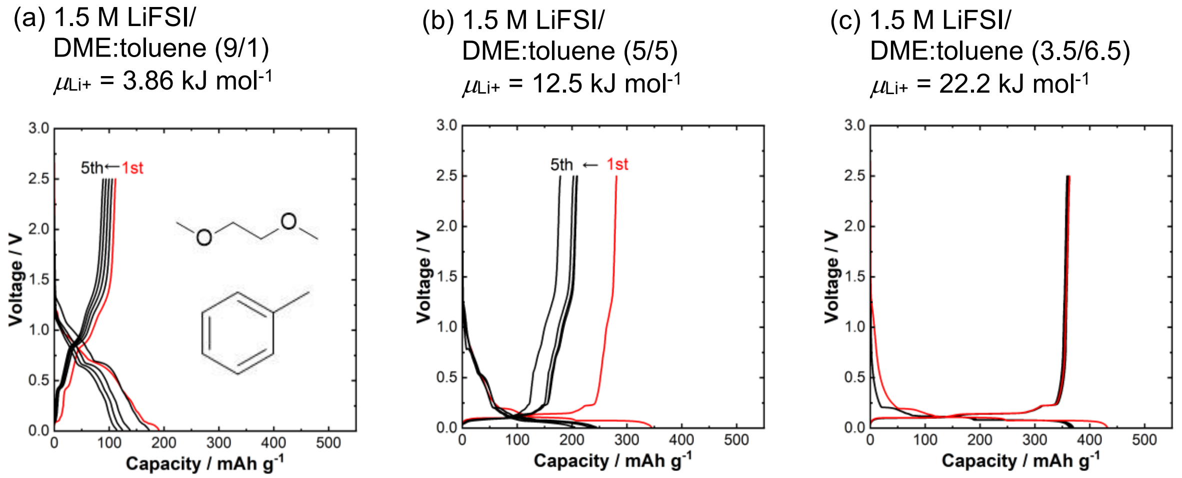

Figure S16. Charge-discharge curves of natural graphite/lithium cells with 1.5 M LiFSI/DME:toluene, n/n ((a)9/1, (b)5/5 and (c) 3.5/6.5 by mol.).at C/10 rate (37.2 mA g^{-1}). The Li^+ chemical potential (μ_{Li+}) with reference to LiFSI/DME (1/10 by mol) is also given.

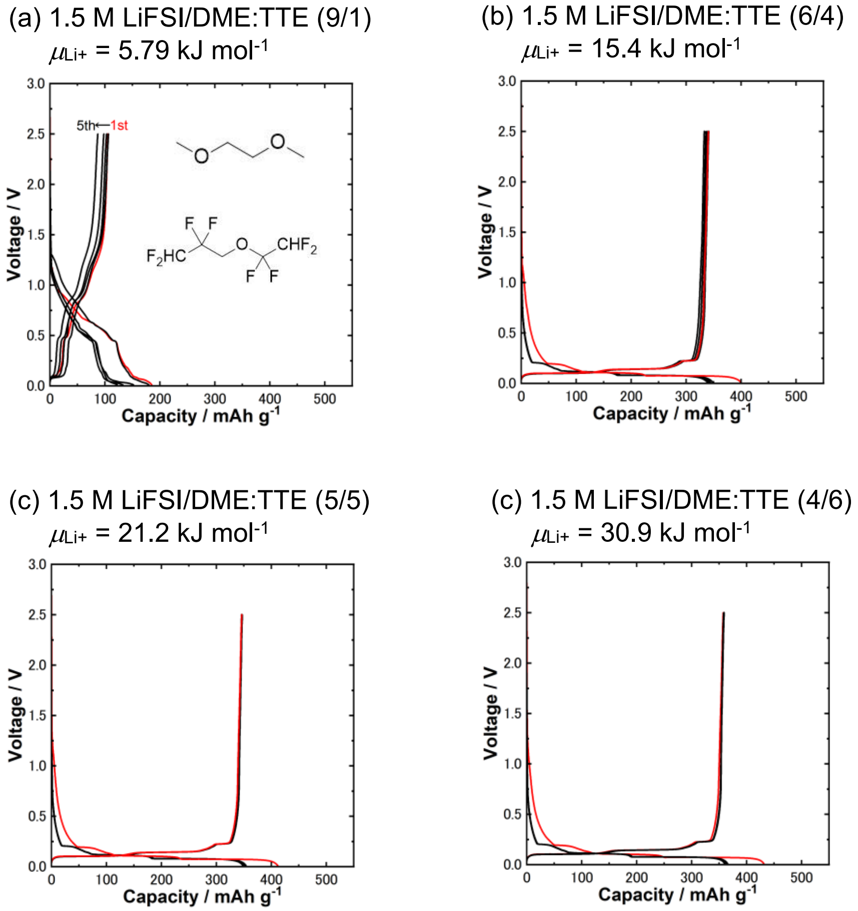

Figure S17. Charge-discharge curves of natural graphite/lithium cells with 1.5 M LiFSI/DME:TTE ((a) 9/1, (b) 6/4, (c) 5/5, and (d) 4/6 by mol) at C/10 rate (37.2 mA g^{-1}). The Li^+ chemical potential (μ_{Li^+}) with reference to LiFSI/DME (1/10 by mol) is also given.

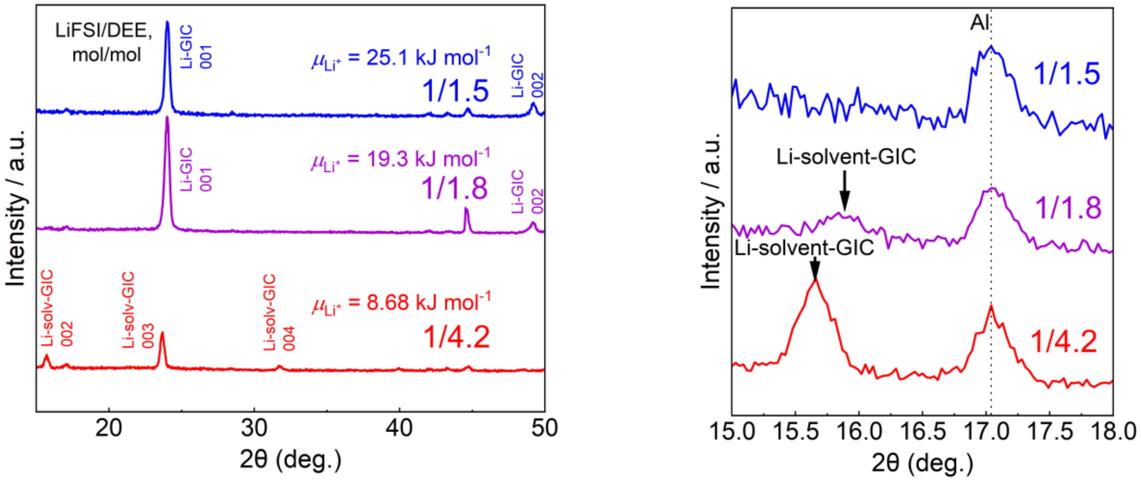

Li-GIC in LiFSI/DEE (1/1.5 and 1/1.8)

2θ	d / nm	00*l*	*lc* / nm
24.04	0.370	001	0.370
49.18	0.185	002	0.370

Li-solvent-GIC in LiFSI/DEE (1/4.2)

2θ	d / nm	00*l*	*lc* / nm
15.69	0.565	002	1.13
23.69	0.376	003	1.13
31.76	0.282	004	1.13

Figure S18. XRD patterns of graphite electrodes discharged to around 0 V vs. Li metal in LiFSI/DEE (1/4.2, 1/1.8, and 1/1.5 by mol). In LiFSI/DEE (1/4.2 by mol), Li-solvent-GIC with an expanded interlayer distance of 1.13 nm was formed. On the other hand, in more concentrated LiFSI/DEE (1/1.5), Li-GIC (LiC_6) with an interlayer distance of 0.37 nm was formed. In the intermediate LiFSI/DEE (1/1.8), Li-GIC (LiC_6) was also formed, but a small peak at $2\theta = 15.8^{\circ}$, as well as sloping voltage curve during charge in 0.5-1.2 V, suggests the formation of Li-solvent-GIC with *Ic* = 1.12 nm.

LiFSI/DEE (1/1.5)
μ_{Li+} = 25.1 kJ mol^{-1}

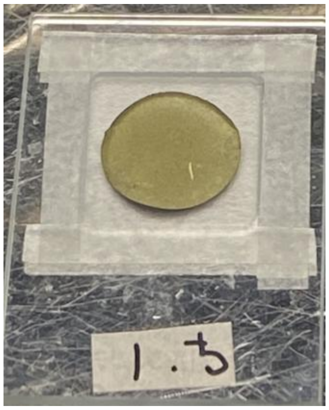

LiFSI/DEE (1/1.8)
μ_{Li+} = 19.3 kJ mol^{-1}

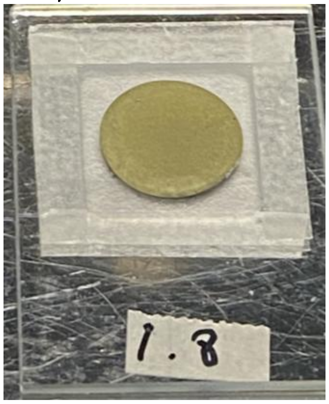

LiFSI/DEE (1/4.2)
μ_{Li+} = 8.68 kJ mol^{-1}

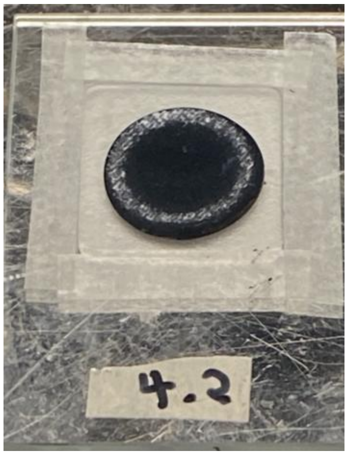

Figure S19. Photo images of graphite electrodes discharged to around 0 V vs. Li metal in LiFSI/DEE (1/1.5, 1/1.8, and 1/4.2 by mol). In LiFSI/DEE (1/1.5 and 1/1.8 by mol), the color was changed to gold, which is characteristic of LiC_6. In LiFSI/DEE (1/4.2 by mol), the electrode becomes thicker and the surface was roughened, which is due to solvent cointercalation.

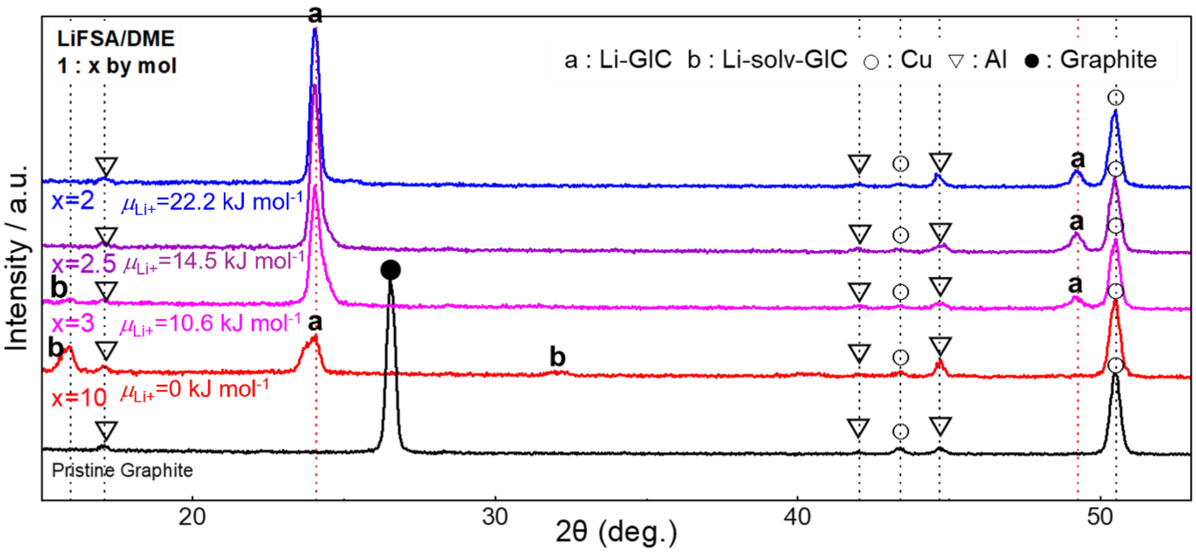

Li-GIC in LiFSI/DME (1/2, 1/2.5, and 1/3)

2θ	d / nm	00*l*	*lc* / nm
24.04	0.370	001	0.370
49.18	0.185	002	0.370

Li-solvent-GIC in LiFSI/DME (1/3 and 1/10)

2θ	d / nm	00*l*	*lc* / nm
15.94	0.556	002	1.11
24.04	0.370	003	1.11
32.22	0.278	004	1.11

Figure S20. XRD patterns of graphite electrodes discharged to around 0 V vs. Li metal in LiFSI/DME (1/10, 1/3, 1/2.5, and 1/2 by mol). In LiFSI/DME (1/10 by mol), Li-solvent-GIC with an expanded interlayer distance of 1.11 nm was formed. On the other hand, in more concentrated LiFSI/DEE (1/2 or 1/2.5), Li-GIC (LiC_6) with an interlayer distance of 0.37 nm was formed. In the intermediate LiFSI/DME (1/3), Li-GIC (LiC_6) was also formed, but a small peak at 2θ = 15.9°, as well as sloping voltage curve during charge in 0.5-1.2 V, suggests the formation of Li-solvent-GIC with *lc* = 1.11 nm.

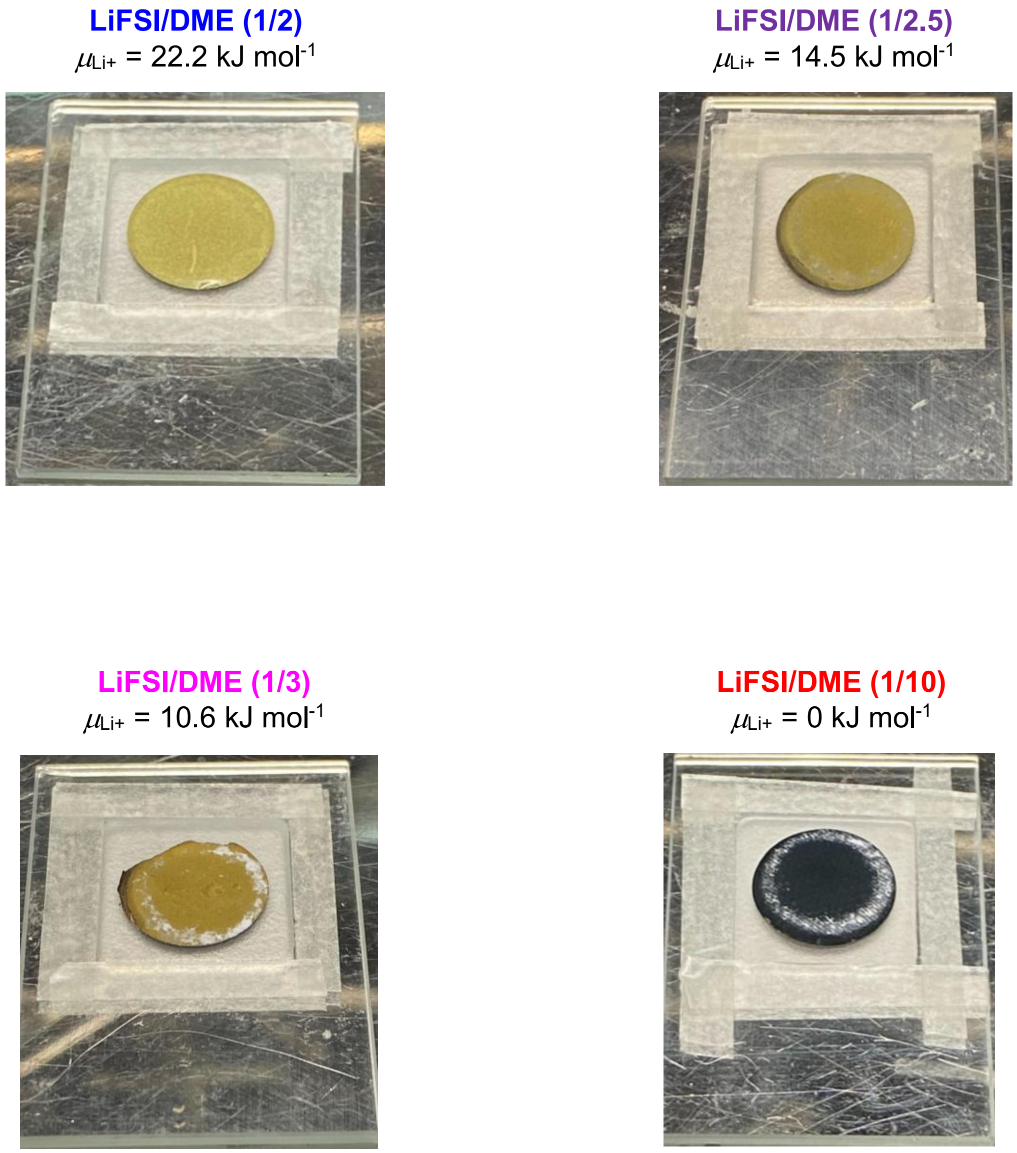

Figure S21. Photo images of graphite electrodes discharged to around 0 V vs. Li metal in LiFSI/DME (1/2, 1/2.5, 1/3, and 1/10 by mol). In LiFSI/DME (1/2 by mol), the color was changed to gold, which is characteristic of LiC_6. In LiFSI/DME (1/2.5 and 1/3 by mol), the gold color became slightly reddish, suggesting the co-existence of another phase (Li-solvent-GIC). In LiFSI/DME (1/10 by mol), the electrode becomes thicker and the surface was roughened, which is due to solvent cointercalation.

(a) LiFSI/DME (1/4)

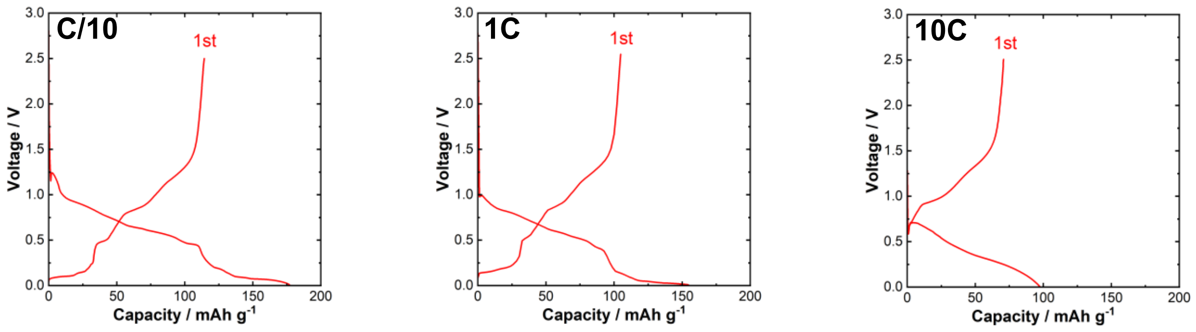

(b) LiFSI/DME (1/3)

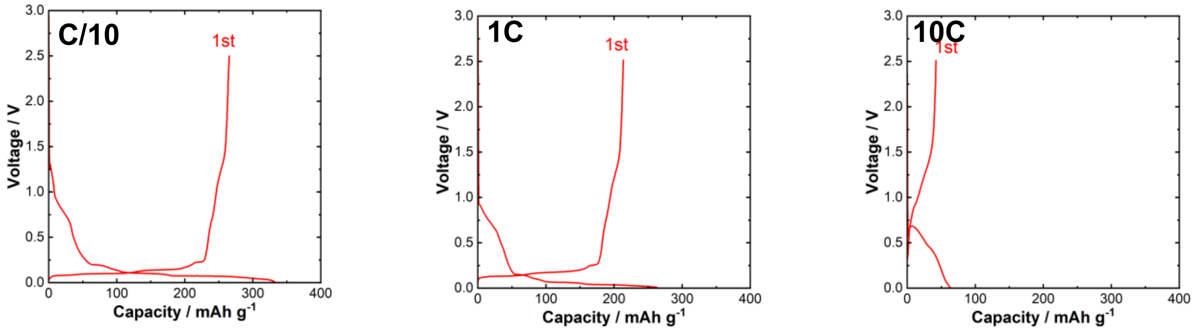

Figure S22. Charge-discharge curves of natural graphite/lithium cells with LiFSI/DME ((a) 1/4 and (b) 1/3 by mol) at C/10, 1C and 10C rates (1C = 372 mA g^{-1}). The solvent cointercalation over 0.5 V still occurred at much higher C rates, suggesting that the solvent cointercalation cannot be suppressed in a kinetic way.

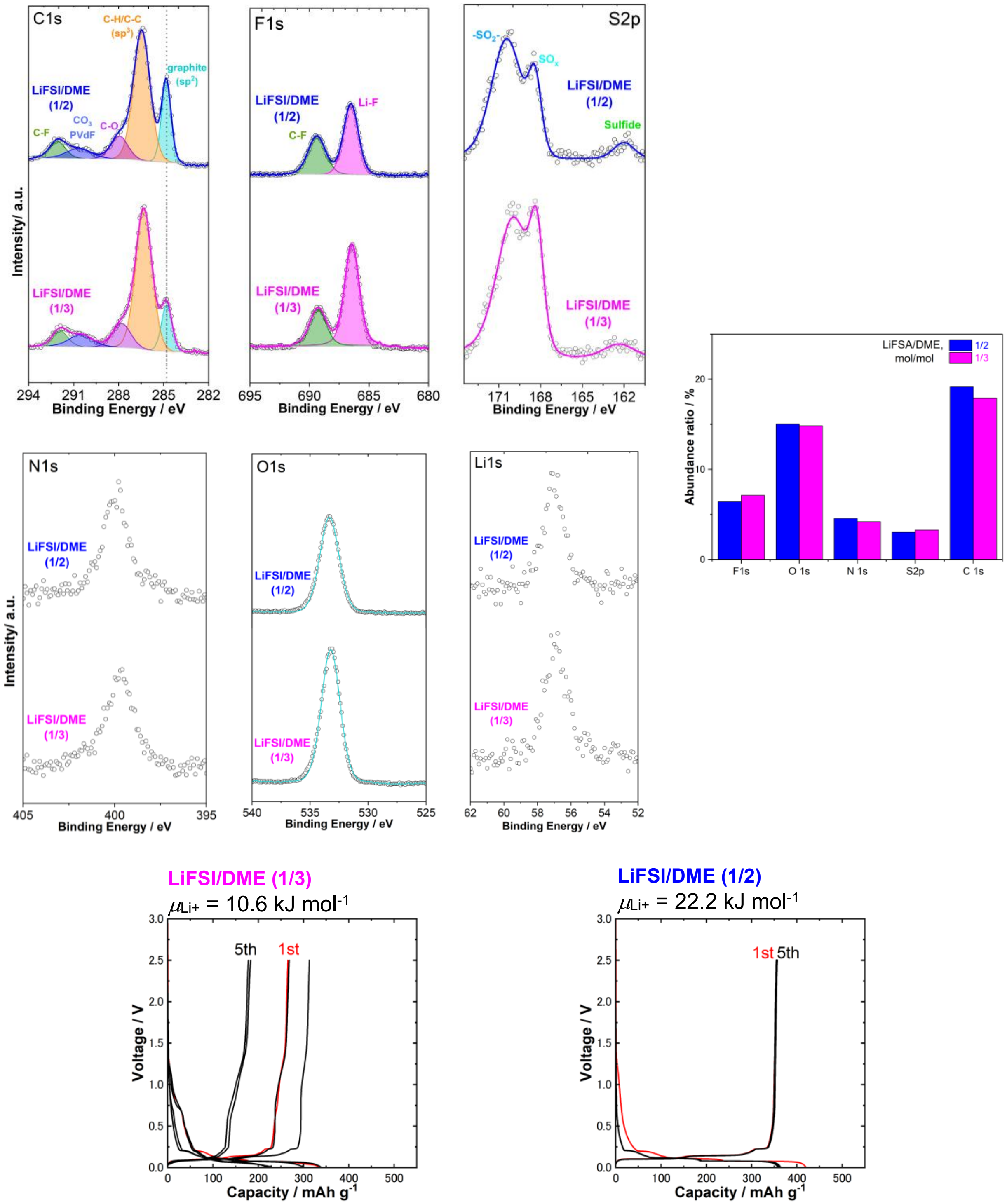

Figure S23. SEI compositions of graphite electrodes after charge-discharge measurements in LiFSI/DME (1/2 and 1/3 by mol) analyzed by XPS.[1] Charge-discharge curves of graphite|Li half cells are also shown. The two electrolytes resulted in remarkably different graphite reactions, but the SEI chemistry was almost the same, suggesting that there is an additional factor that dominate the graphite reactions.

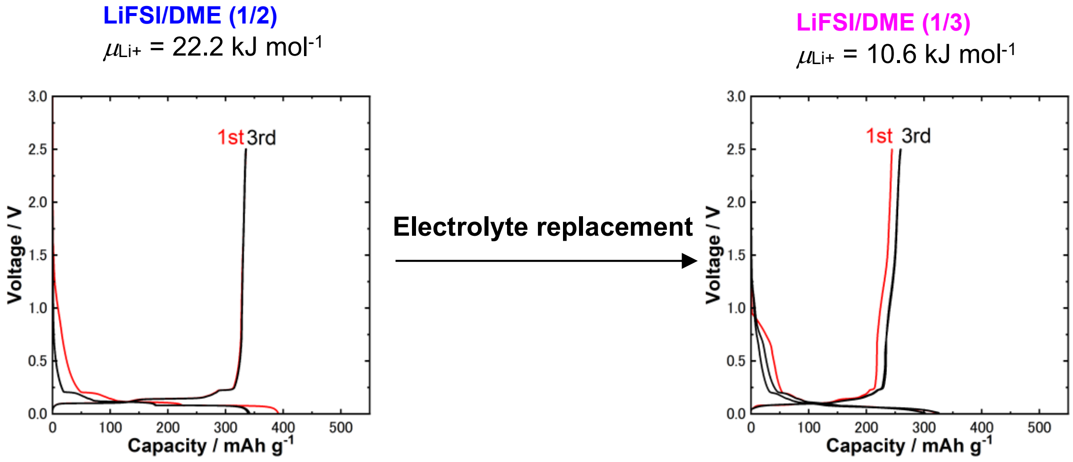

Figure S24. Charge-discharge curves of graphite|Li half cell with LiFSI/DME (1/2 by mol) and subsequent cycles after replacing the electrolyte for LiFSI/DME (1/3 by mol). Solvent cointercalation could not be suppressed effectively in LiFSI/DME (1/3 by mol) even after forming anion-derived SEI on graphite in the higher-μ_{Li^+} electrolyte, LiFSI/DME (1/2 by mol).

References

1. Y. Yamada, K. Furukawa, K. Sodeyama, K. Kikuchi, M. Yaegashi, Y. Tateyama, A. Yamada, "Unusual Stability of Acetonitrile-Based Superconcentrated Electrolytes for Fast-Charging Lithium-Ion Batteries" *J. Am. Chem. Soc.* **2014**, *136*, 5039.
